# Webcams as Windows to the Mind? A Direct Comparison Between In-Lab and Web-Based Eye-Tracking Methods

**DOI:** 10.1162/opmi_a_00171

**Published:** 2024-11-22

**Authors:** Mieke Sarah Slim, Margaret Kandel, Anthony Yacovone, Jesse Snedeker

**Affiliations:** Language Development Department, Max Planck Institute for Psycholinguistics, Nijmegen, Netherlands; Department of Experimental Psychology, Ghent University, Ghent, Belgium; Department of Psychology, Harvard University, Cambridge, MA, USA; Department of Linguistics, Boston University, Boston, MA, USA

**Keywords:** eye-tracking, web-based experimentation, real-time processing, visual world paradigm, language processing, psycholinguistics, WebGazer

## Abstract

There is a growing interest in the use of webcams to conduct eye-tracking experiments over the internet. We assessed the performance of two webcam-based eye-tracking techniques for behavioral research: manual annotation of webcam videos (*manual eye-tracking*) and the automated WebGazer eye-tracking algorithm. We compared these methods to a traditional infrared eye-tracker and assessed their performance in both lab and web-based settings. In both lab and web experiments, participants completed the same battery of five tasks, selected to trigger effects of various sizes: two visual fixation tasks and three visual world tasks testing real-time (psycholinguistic) processing effects. In the lab experiment, we simultaneously collected infrared eye-tracking, manual eye-tracking, and WebGazer data; in the web experiment, we simultaneously collected manual eye-tracking and WebGazer data. We found that the two webcam-based methods are suited to capture different types of eye-movement patterns. Manual eye-tracking, similar to infrared eye-tracking, detected both large and small effects. WebGazer, however, showed less accuracy in detecting short, subtle effects. There was no notable effect of setting for either method. We discuss the trade-offs researchers face when choosing eye-tracking methods and offer advice for conducting eye-tracking experiments over the internet.

## INTRODUCTION

Eye-tracking is widely used by behavioral researchers to study real-time, moment-to-moment processing (e.g., Holmqvist et al., 2015; Just & Carpenter, 1976). While eye-tracking studies have traditionally taken place in labs using specialized eye-tracking equipment, researchers have recently sought out alternative methods to collect eye-tracking data over the internet using consumer-grade equipment like webcams. These webcam eye-tracking methods use video recordings to estimate where the participant is looking, either by using automated gaze-estimation algorithms (e.g., *WebGazer*; Papoutsaki et al., 2016) or by hand-annotating gaze direction from recorded videos. This latter technique, which we will refer to as *manual eye-tracking*, has previously been applied in lab settings (e.g., Snedeker & Trueswell, 2004; Trueswell, 2008; Yacovone et al., 2021; sometimes called *poor-man’s eye-tracking*). The benefits of web-based research methods using off-the-shelf hardware are obvious: by programming an experiment that participants can complete from wherever they are, researchers can recruit a much larger and more diverse sample than is feasible in lab-based studies (e.g., Hartshorne et al., 2018; Li et al., 2024). In addition, there is no need for expensive hardware, and these techniques involve equipment that is easily portable (a laptop with a webcam), thereby additionally allowing researchers to recruit participants in settings such as museums, schools, or field sites.

However, webcam eye-tracking methods may be less accurate than high-end eye-tracking devices, especially when used in web-based settings (e.g., Semmelmann & Weigelt, 2018). For example, in language processing research, classic psycholinguistic eye-movement effects appear to be delayed and noisier in experiments using WebGazer relative to in-lab effects (e.g., Degen et al., 2021; Kandel & Snedeker, 2024; Slim & Hartsuiker, 2023). Apparent temporal delays and reduced spatial accuracy have also been observed using manual eye-tracking with webcam video recordings (Ovans, 2022). This raises important concerns about the efficacy of these techniques for behavioral research investigating real-time cognitive processing, where shifts in the timing of an effect often have implications for the theoretical conclusions that we can draw (e.g., Slim & Hartsuiker, 2023).

We cannot unambiguously identify the source of this reduced spatiotemporal accuracy: Is it due to noise introduced by conducting the research in a less-controlled web-based setting? Does it result from properties of the webcam eye-tracking methods themselves? Or is it due to a combination of these two factors? The fact that participants complete the experiment outside of the lab using their own devices introduces additional noise in the data (due to variation across participants in hardware, software, internet connections, ambient lighting conditions, external distractions, etc.; e.g., Semmelmann & Weigelt, 2018). The cumulative effect of these various factors could be a reduction in the signal-to-noise ratio resulting in it taking longer for a given eye-movement pattern to become detectable. Given that most of recent web-based eye-tracking studies have used an automatic gaze-coding algorithms, typically WebGazer (e.g., Degen et al., 2021; Semmelmann & Weigelt, 2018; Slim & Hartsuiker, 2023; Vos et al., 2022; Yang & Krajbich, 2021), apparent delays could also result from the time-consuming execution of the gaze estimation algorithm and/or increased variation in the data due to inaccuracy in estimation.

In the present study, we compare three distinct eye-tracking methods: a specialized in-lab infrared eye-tracker (a screen-based Tobii T60), the WebGazer algorithm, and manual eye-tracking using recorded webcam videos. Our study is the first to simultaneously record data with these three different methods, which allows us to do a systematic comparison of data collected within the same session, holding participant behavior and environment constant. We characterize the differences in the spatial and temporal accuracy of these methods in a battery of five tasks designed to elicit eye-movement patterns of the type relevant to behavioral researchers (and language scientists in particular). The tasks include simple visual fixation tasks in addition to language-based tasks (using the *visual world paradigm*; more below). Altogether, these tasks provide an ideal testing ground for assessing the methods’ ability to detect both (i) basic eye-movement patterns and (ii) moment-to-moment processing of temporally-unfolding stimuli (i.e., words and sentences). In addition, to assess the influence of web-based testing on eye-movement data, we conducted versions of the experiment both in the lab (using all three eye-tracking methods) and over the internet (using the two webcam-based methods). With this study design, we are able to assess the data quality of each eye-tracking method as well as the role of web-based testing, allowing us to disentangle the source of the noise observed in prior webcam-based eye-tracking experiments.

In the remainder of this Introduction, we first describe the two webcam-based eye-tracking techniques in more detail and then end by introducing the various tasks that we used in the present study.

### Automated Webcam-Based Eye-Tracking

In recent years, behavioral science has seen a growing interest in automated eye-tracking methods that rely on consumer-grade webcams to capture videos of the participant’s eyes (e.g., Erel et al., 2023; Papoutsaki et al., 2016; Valenti et al., 2009; Valliappan et al., 2020; Xu et al., 2015). The most widely-used method in behavioral research is currently *WebGazer*, an open-source JavaScript library that can be used to collect eye-tracking data within a web browser (Papoutsaki et al., 2016). WebGazer consists of two components: (i) face detection software that estimates the position of the pupils in the webcam stream (relative to the participant’s face) and (ii) a gaze estimator algorithm that estimates the participant’s gaze location on the computer screen. This gaze estimator is calibrated through mouse click interactions in the browser, as gaze typically follows cursor movements (Chen et al., 2001; Huang et al., 2012; Papoutsaki et al., 2016, 2018).

WebGazer (and similar automated eye-tracking methods using webcams) functions differently from specialized eye-tracking devices used in lab-based experiments. These latter devices shine near-infrared light (invisible to the human eye) towards the participant’s cornea. The corneal reflection is then captured by a high-end camera. Video processing algorithms estimate the participant’s gaze location on the screen by locating the pupil in relation to the corneal reflection (e.g., Tobii Pro, 2021; SR Research, 2021). The eye-tracking data are then recorded and saved as coordinates on the screen. These specialized devices (which we refer to as *infrared eye-trackers*) are very accurate (both in the temporal and in the spatial domains; e.g., Ehinger et al., 2019; Nyström et al., 2021), but they are relatively expensive and not always easily portable. This makes them less suitable for certain research teams and purposes.

In contrast, WebGazer is free to use and can be implemented wherever one has access to a consumer-grade computer, a webcam, and an internet connection (if the participant is tested over the web). WebGazer runs fully in the browser, so it is not necessary to install additional software. Furthermore, like infrared eye-trackers, WebGazer saves gaze locations in the form of screen coordinates. Moreover, WebGazer has been incorporated into several popular experiment builders (e.g., *Gorilla*, *jsPsych*, *PsychoPy*, and *PCIbex*; Anwyl-Irvine et al., 2020; de Leeuw, 2015; Peirce, 2007; Zehr & Schwarz, 2018), which makes it easy to implement WebGazer in web-based behavioral experiments.

In one of the first validation studies of WebGazer for behavioral science, Semmelmann and Weigelt (2018) assessed how well WebGazer detects simple eye movements using a fixation task and a smooth pursuit task. They found that gaze settles on visual stimuli roughly 800–1000 ms after stimulus onset and that the spatial offset between estimated target fixations and the stimuli was roughly 4–10° of the visual angle (see also Slim & Hartsuiker, 2023, for similar results). For comparison, infrared eye-trackers typically detect similar changes in fixation less than 200 ms after stimulus offset (Ehinger et al., 2019) and have spatial offsets of less than 0.5° (e.g., Dalrymple et al., 2018; Ehinger et al., 2019). Importantly, Semmelmann and Weigelt (2018) observed differences in WebGazer data quality when the same experiment was run in the lab and remotely over the internet: the looking patterns in the web-acquired data appeared slower, and the spatial distance between the estimated gaze locations and stimuli was larger. The authors speculated that these differences between lab- and web-based testing are caused by variation in hardware used in the web-based experiment and by the decreased control of the environment in the web-based setting (Semmelmann & Weigelt, 2018).

In addition to assessing WebGazer’s performance detecting simple eye movements, several recent studies have further assessed WebGazer’s ability to replicate eye-tracking effects in behavioral science (e.g., Degen et al., 2021; Kandel & Snedeker, 2024; Semmelmann & Weigelt, 2018; Slim & Hartsuiker, 2023; Vos et al., 2022; Yang & Krajbich, 2021). For instance, Semmelmann and Weigelt (2018) successfully replicated a well-established eye-movement pattern in face inspection, finding that their participants mostly looked at the eyes when presented with images of faces (although the size of the effect was smaller in the web-acquired than in the lab-acquired data).

Other studies have tested WebGazer’s ability to detect temporally-sensitive eye-tracking effects indicative of real-time cognitive processing. For example, Slim and Hartsuiker (2023) tested the efficacy of WebGazer to detect eye-movement patterns associated with real-time cognitive processing using a visual world paradigm. In this paradigm, participants are shown displays of visual stimuli (typically with one picture in each quadrant of the display) as they hear a linguistic cue. The paradigm takes advantage of the tight temporal link between visual attention and language processing to determine how participants are processing these cues in real-time (see Huettig et al., 2011, for review). Specifically, Slim and Hartsuiker (2023) focused on the phenomenon of *verb-mediated anticipatory looking* (e.g., Altmann & Kamide, 1999; Dijkgraaf et al., 2017): upon hearing a semantically-constraining verb, listeners predictively look to images depicted on the screen that are suitable objects for the verb, even before hearing the object name in the sentence. For instance, when hearing the sentence *Mary tastes a carrot* while looking at a display containing a carrot, a dress, a napkin, and a ruler, participants look at the picture of the carrot (the only edible object in the display) while hearing the verb *taste*. In contrast, if the verb is consistent with all of the objects on the screen (e.g., *Mary grabs a carrot*), then participants do not look at the picture of the carrot until they hear its name in the sentence (Dijkgraaf et al., 2017).

While Slim and Hartsuiker (2023) observed the expected timing difference in looks to target images in sentences with constraining and non-constraining verbs, this effect was smaller and started approximately 200 ms later than when the same experiment was run in-lab using infrared eye-tracking (Dijkgraaf et al., 2017). Crucially, this 200 ms delay meant that the relevant effect could not be detected until *after* the object noun onset, and thus, if we had to interpret the Slim and Hartsuiker (2023) data in isolation, we would not be able to safely conclude that the effect is due to *anticipatory* processing. Similar reductions in effect sizes and delays in effect onsets relative to in-lab studies have been observed in other studies using WebGazer (e.g., Degen et al., 2021; Kandel & Snedeker, 2024). In short, the reduced temporal resolution of WebGazer is potentially problematic in studies of *real-time* processing, and understanding the nature of this delay is critical for integrating evidence across methods ensuring replicable findings and a cumulative science.

Delays in the effect timing relative to in-lab studies may result from several sources. Firstly, the WebGazer algorithm could introduce a time lag itself: the execution speed of WebGazer may not be fast enough to pick up changes in fixation in the real-time (especially if the processing demand on the browser is high; see also Vos et al., 2022; Yang & Krajbich, 2021). Secondly, web-based settings introduce additional noise to the data (Semmelmann & Weigelt, 2018), which could make the initial onsets of effects more difficult to discriminate, leading to detection of effects only if eye-movement patterns are sufficiently large (e.g., Kandel & Snedeker, 2024). In the present study, we aim to disentangle these two sources of noise by comparing the performance of WebGazer in a lab-based and a web-based setting. Moreover, we will gain insight into potential time lags introduced by WebGazer by comparing WebGazer’s performance to the performance of another webcam-based eye-tracking technique: manual eye-tracking.

### Manual Eye-Tracking

While WebGazer is a relatively new technique for collecting eye-tracking data with off-the-shelf hardware, *manual* eye-tracking has been used in research labs for over two decades (e.g., Snedeker & Trueswell, 2004). This method uses consumer-grade video equipment to record a participant’s face while they do the experiment. The video recording is then time-locked to the experimental trials, and a trained coder (who is naive to the experimental condition of each trial) manually annotates the participant’s gaze direction by stepping through the video frame-by-frame. This method thus differs from automated gaze-recording methods, like infrared eye-tracking and WebGazer, which only record gaze locations and do not save any images of the participant’s face.

This technique has been successfully applied in the lab with video cameras to detect the same types of eye-tracking effects detected by infrared eye-trackers (e.g., Huang & Snedeker, 2009; Qi et al., 2011; Snedeker & Trueswell, 2004; Thothathiri & Snedeker, 2008; Yacovone et al., 2021). Moreover, in a small visual world validation study (*n* = 2), Snedeker and Trueswell (2004) compared simultaneously-collected data from a specialized head-mounted eye-tracking device and manual eye-tracking and found that both methods provided very similar data: in all recorded frames, there was over 90% agreement in gaze location. Altogether, these findings suggest that the timing of effects detected using manual eye-tracking is comparable to those observed when using specialized eye-trackers. However, the spatial resolution of manual eye-tracking is inherently different from infrared eye-tracking, as estimated gaze locations are defined as looks to regions of the screen (e.g., top-left quadrant, top-right quadrant, center, and so on) as opposed to the precise coordinates estimated by infrared eye-trackers.

Researchers have recently investigated whether manual eye-tracking can be carried out remotely over the internet, using the participants’ own webcams to make video recordings as participants complete a visual world experiment (Kandel & Snedeker, 2024; Ovans, 2022). In an early validation study, Ovans (2022) used this method in two visual world experiments. One experiment tested the robust verb-mediated anticipatory looking effect in sentence processing (also tested by Slim & Hartsuiker, 2023; described above). The second experiment tested the *phonemic cohort* and *rhyme competition* effects in word processing (both previously observed in Allopenna et al., 1998). In the phonemic cohort effect, as participants hear a target word (e.g., *beaker*), they look more at images whose names start with the same onset sounds as the target (e.g., beetle) than they do at unrelated distractor images (e.g., carriage). In the rhyme effect, participants look more at rhyme competitors of the target (e.g., *speaker* – *beaker*) than unrelated distractors (Allopenna et al., 1998). In both experiments, the browser automatically recorded webcam videos during each trial (using PCIbex software; Zehr & Schwarz, 2018), which were later used for hand annotation.

Ovans’ (2022) experiments successfully replicated the verb-mediated anticipatory looking effect and the phonemic cohort effect. However, they did not replicate the rhyme effect, which is smaller than the phonemic cohort effect (e.g., Allopenna et al., 1998). In a similar study, Kandel and Snedeker (2024) replicated the phonemic cohort effect using manual eye-tracking of webcam videos with both adult and child participants (5–6 years-old). In this study, participants completed the experiment while in a *Zoom* teleconferencing video call with the experimenter(s) (https://zoom.us), and participant webcam videos were collected via the Zoom meeting recording function for later annotation. Thus, manual eye-tracking can not only successfully be applied to detect behavioral eye-tracking effects in web-based settings, but there is also flexibility in the way researchers can record webcam videos for annotation in their experiments.

At first glance, manual eye-tracking with webcam videos appears to have better spatial and temporal accuracy for detecting looks to a given quadrant than WebGazer. In Ovans’ (2022) verb-mediated anticipatory looking experiment, the effect was detected in the expected time window (prior to the onset of the post-verbal noun), contrary to what was observed by Slim and Hartsuiker (2023) using WebGazer. Furthermore, in a direct comparison of webcam video annotations and WebGazer gaze estimates from the same participants, Kandel and Snedeker (2024) observed earlier and larger eye-movement patterns in the webcam video data.

However, although manual eye-tracking appears to yield higher-quality data than WebGazer, it at times appears noisier than in-lab infrared eye-tracking. The onset of the phonemic cohort effect in Ovans (2022) was delayed by approximately 200 ms compared to that observed in the lab by Allopenna et al. (1998), and the proportion of detected looks to the target images in the task was lower (<75% vs. >90%). Moreover, Ovans’ (2022) study did not replicate the more subtle rhyme effect. It thus appears that the timing of effects detected using webcam manual eye-tracking can be slower than expected in infrared eye-tracking, and subtle, fleeting effects (like the rhyme effect) may not steadily emerge.

No study so far has systematically compared manual eye-tracking to both WebGazer and infrared eye-tracking. A systematic comparison of these methods (with simultaneously-collected data, more below) will allow us to better understand the relative strengths and weaknesses of each method.

### The Present Study

In the present study, we simultaneously collect data from three different eye-tracking methods in order to systematically compare the methods’ suitability for behavioral research. We compare eye-movement data obtained using: (i) infrared eye-tracking with a Tobii T60, (ii) manual eye-tracking of webcam videos recorded using Zoom teleconferencing software, and (iii) WebGazer. Moreover, we collect data from the two webcam-based methods (WebGazer and manual eye-tracking) in both an in-lab experiment (the *lab experiment*) and a web-based experiment (the *web experiment*), allowing us to see the influence of experimental setting on the eye-movement data.

We chose to use Zoom for the webcam video recordings for several reasons: (i) Zoom is a popular, freely-available teleconferencing software that many people likely have already installed on their computers, (ii) Zoom offers a variety of meeting recording layouts, which allowed us to record the participant’s face and screen in separate recordings, and (iii) Zoom standardizes the frame rate of the webcam while recording video (see Method Sample Rates section), which allowed us to process all video data in a uniform way. Because Zoom is teleconferencing software, we could conduct the web experiment in a *supervised* setting: the participants took part in the experiment from their respective locations (using their own devices) while on a Zoom telecall with an experimenter. We chose this supervised testing procedure to increase the similarity of the experimental context in the lab and web experiments. Many web-based experiments (including most of the webcam eye-tracking experiments cited so far) are *unsupervised*, meaning that no experimenter is present at the time of participation (virtually or otherwise). By using a supervised procedure, we hope to unravel possible noise introduced by the web-based nature of the task (that is, the influence of environment and hardware differences) from noise introduced by unsupervised testing (e.g., misunderstandings of the task or set-up procedure or inattentiveness because there is no direct supervision).

In both our lab and web experiments, the participants completed the same battery of five tasks (Table 1). The task battery included a fixation task, a smooth pursuit task, a lexical fixation task, a phonemic cohort task, and an anticipatory looking task. These tasks were selected to test basic properties of eye movements (the fixation and smooth pursuit tasks) as well as well-known eye-movement patterns and effects indicative of real-time language processing (the lexical fixation, phonemic cohort, and anticipatory looking tasks). The behavioral effects we chose to replicate are robust language processing effects elicited using the visual world paradigm. This paradigm is useful for assessing the eye-tracking methods’ suitability for studying real-time cognitive processing, as the gaze patterns it prompts can involve a combination of both robust eye-movements (target looks) as well as more sensitive effects (e.g., the phonemic cohort effect).

**Table 1.** Overview of the Experimental Task Battery (in Order of Presentation)

**Task**	**Description**
Fixation	Participants look at circles that appear in the quadrants of the screen.
	**Goal:** Assess saccade detection and accuracy of target looks in response to visual cues.
Smooth pursuit	Participants follow a circle with their gaze as it moves around the screen.
	**Goal**: Assess the spatiotemporal offset in estimated gaze locations compared to the true location of the stimulus (infrared eye-tracking and WebGazer only).
Lexical fixation	Participants see images in each screen quadrant (the images remain fixed throughout the task), and in each trial, they hear the name of an image to look at.
	**Goal**: Assess saccade detection and accuracy of target looks in response to real-time processing of linguistic cues.
Phonemic cohort	In each trial, participants are instructed to look at one of four images displayed on the screen (the target). We manipulate whether there is an image in the display (the competitor) whose name shares onset sounds with the target.
	**Goal**: Assess the sensitivity to detect a spatiotemporally-sensitive real-time language processing effect at the word level (the phonemic cohort effect).
Anticipatory looking	In each trial, participants see four images and select the image that goes with an auditorily-presented sentence. Two images are related to the sentence subject (the agent). We manipulate whether the verb is semantically constraining or non-constraining. In the constraining condition, one image (the target) is related to both the agent and the verb, making it a predictable object of the verb. In the non-constraining condition, all images are in principle suitable verb objects.
	**Goal:** Assess the sensitivity to detect anticipatory looks to the agent-related images and the target image (indicative of real-time language processing at the sentence level).

We first describe the experimental apparatus and set-up for both the lab and the web experiment as well as the general data analysis procedure used in the study. We then present the study results by task. For each task, we provide the task description, specific analyses, results, and a results summary. More detailed methods for each task are available in the Supplementary Materials (section A. Detailed Methods). The methods and analyses for both experiments and their comparison were preregistered. The Supplementary Materials, preregistrations, data, and analysis code for the present study are available on OSF: https://osf.io/sk8wu/.

## METHODS

In the lab experiment, we directly compared the performance of (i) a Tobii T60 infrared eye-tracker, (ii) manual eye-tracking using webcam video recordings, and (iii) WebGazer as participants completed the task battery in the lab. The three eye-tracking methods recorded gaze data simultaneously within each session, allowing for a direct comparison.

The web experiment replicated the lab experiment in a web-based setting. We simultaneously collected (i) webcam videos for manual eye-tracking and (ii) WebGazer data, allowing for a direct comparison of these methods in a web-based setting. By comparing the lab and web experiments, we are also able to assess the influence of the web-based setting on the performance of the two webcam-based eye-tracking methods.

### Participants

#### Lab Experiment.

Our lab experiment sample consisted of 32 native speakers of American English for whom American English was either their only or their dominant language (*M*_age_ = 21.9 years, *SD*: 5.8 years, range: 8–43 years; 18 F, 14 M). Due to technical issues in the processing of the manual eye-tracking data (which will be described below; see Data Exclusion section), we were unable to analyze the manual eye-tracking for 12 participants. In the individual analyses of each dataset (infrared eye-tracking, WebGazer, and manual eye-tracking), we included the data from the 20 participants who had analyzable data for all eye-tracking methods.^1^ We report relevant differences in the infrared eye-tracking and WebGazer analyses when using the full sample of participants. Analyses comparing across datasets (see Analysis Procedure section) included all participants with analyzable data of the relevant types.

An additional five participants completed the lab experiment but were omitted from the sample due to technical issues with the Tobii Pro Studio gaze recording software used by the Tobii T60 eye-tracking device (*n* = 1) or because American English was not their dominant language (*n* = 4). Any additional participants omitted from task analyses are reported with each task in Tasks and Results section.

The participants were recruited from the Harvard University Psychology Study Pool (SONA) or via the researchers’ personal networks. They received either course credit or payment ($10) for their participation.

#### Web Experiment.

The web experiment sample consisted of 42 native speakers of American English for whom American English was either their only or their dominant language (*M*_age_ = 30.1 years, *SD*: 9.5 years, range: 18–58 years; 23 F, 18 M, 1 preferred not to say). Although our preregistered target sample size was the same as the lab experiment (*n* = 32), technical issues in the processing of the manual eye-tracking data (see Data Exclusion section) made this data unanalyzable for 22 participants. We consequently collected data from an additional 10 participants in order to balance the number of participants with analyzable data for all methods between the lab and web experiments. As in the lab experiment, in the individual analyses of each dataset (WebGazer and manual eye-tracking), we included the data from the 20 participants who had analyzable data for all eye-tracking methods.^2^

An additional three participants completed the web experiment but were omitted from the sample because American English was not their native or dominant language. Any additional participants omitted from task analyses are reported with each task in Tasks and Results section.

The participants were recruited from the Harvard University Psychology Study Pool (SONA) or via word of mouth within the researchers’ personal networks. For their participation, participants were compensated with either course credit or payment (a $10 gift card).

### Experimental Apparatus

#### Lab Experiment.

The lab experiment was presented to participants on a Tobii T60 screen-based eye-tracker connected to an Acer Aspire 5 (A515-54-51DJ) laptop (running Microsoft Windows 11). Two Logitech C920 webcams were mounted on top of each other above the (17 inch) Tobii screen (see Supplementary Materials, Figure 1). One of these webcams recorded data using WebGazer, and the other webcam recorded the participant’s face via Zoom teleconferencing software. We used two webcams for data collection because Windows 11 does not allow simultaneous use of the webcam by multiple applications. We counterbalanced between participants and conditions whether the top or bottom webcam was used for WebGazer or Zoom.

The experiment was implemented in *PennController for IBEX* v2.0 (*PCIbex*) and hosted on the *PCIbex Farm* (Zehr & Schwarz, 2018). PCIbex is a JavaScript-based software library for building browser-based experiments. Crucial for our purposes, PCIbex contains an *eyetracker* element, which invokes the WebGazer algorithm to estimate gaze locations using the webcam stream (Papoutsaki et al., 2016). The experiments used the PCIbex implementation of WebGazer available at the initial time of testing (June–December 2022). In PCIbex, the sizes and locations of visual stimuli were defined based on proportions of the display size, which allows the experiment to scale with screen size (see Supplementary Materials, section A. Detailed Methods). This function is relevant because we used the same PCIbex experiment for both the lab experiment (where participants all saw the experiment on the same 17 inch Tobii T60 screen) and the web experiment (where participants completed the experiment on their own devices, which had a variety of screen sizes). The fact that the experiment scaled by screen size ensured that the experiment looked similar across different displays.

The experiment ran in the Mozilla Firefox browser, while two programs ran in the background: *Tobii Pro Studio* v3.4.8 and *Zoom* (https://zoom.us). We used Tobii Pro Studio’s screen recording function to record data with the Tobii infrared eye-tracker. This function captured eye movements continuously as the participant completed the experiment. It also generated a video of the Tobii screen display. We used Zoom’s meeting recording function to make continuous video recordings of the participant’s face and of the screen.

Despite the in-lab nature of this experiment, we presented the experiment in the browser (rather than using offline stimulus presentation software) in order to keep our lab experiment similar to our web experiment and to previous web-based studies (Degen et al., 2021; Kandel & Snedeker, 2024; Semmelmann & Weigelt, 2018; Slim & Hartsuiker, 2023).

#### Web Experiment.

Participants completed the web experiment from their own devices. Participants were required to complete the experiment from a Mac computer: Mac OS allows simultaneous use of one webcam by multiple programs (in our case, by WebGazer and Zoom), while many other computer operating systems (e.g., Microsoft Windows) do not. Participants used a variety of MacBook Pro (*n* = 20), MacBook Air (*n* = 20), and iMac (*n* = 2) devices ranging from 2012 models to 2023 models. Screen sizes ranged from 13.3 inches to 27 inches (see Supplementary Materials, Table 1). All participants used the built-in webcams that came with their computers.

Participants completed the PCIbex experiment (which was the same as in lab experiment) in either the Mozilla Firefox (*n* = 1) or Google Chrome (*n* = 41) web browser. We restricted participants to using one of those two web browsers, because PCIbex’s implementation of WebGazer did not run correctly in other browsers (e.g., Safari) at the time of data collection. We allowed participants to select their preferred browser to complete the experiment.

The participants received instructions on how to set up the experiment while on a Zoom teleconferencing call with an experimenter. As the participant completed the tasks, Zoom continued to run in the background and made a video recording of the participant’s face and of the screen.

#### Method Sample Rates.

The Tobii infrared eye-tracker had a sample rate of 60 frames per second (*fps*). Given that WebGazer sample rates can vary (e.g., Kandel & Snedeker, 2024; Semmelmann & Weigelt, 2018; Slim & Hartsuiker, 2023), we estimated the WebGazer sample rates in our experiments by calculating the average fps rate for each participant in each task. Then, we took the grand mean of those values for each participant. In the lab experiment, the grand mean participant WebGazer sample rate was 12 fps (*SD*: 0.4, range: 11–13). In the web experiment, the grand mean participant WebGazer sample rate was 26 fps (*SD*: 8.9, range: 9–39). The increased variation in the WebGazer sample rate in the web experiment is likely attributable to differences in participant set-ups (all lab participants completed the experiment on the same set-up). The higher WebGazer sample rate in the web experiment compared to the lab experiment could result from webcam or computer processing speed/load differences between web participant set-ups and the lab experiment set-up. Zoom webcam videos were recorded at a standardized sample rate of 25 fps.

### Procedure

#### Lab Experiment.

Participants in the lab experiment were seated centrally in front of the Tobii screen. After the participant gave their informed consent, the experimenter used Zoom to start a recording of both the screen and of the participant’s face. Any Zoom controls and windows were hidden from the screen. Then, the experiment began with a brief (5-point) calibration procedure in Tobii Pro Studio to calibrate the infrared eye-tracker. After calibrating and initiating the infrared eye-tracker, the participant started the experiment in PCIbex. The infrared eye-tracker recorded both the screen and the participant’s gaze data throughout the duration of the experiment.

The experiment on PCIbex was completed in full screen. The experiment started with a brief audio check to ensure that the audio volume was set up correctly (and to allow for adjustments as needed). The audio check was followed by a WebGazer calibration procedure. In this calibration procedure, the participant fixates on eight circles that appear one-by-one in a random order along the edges of the screen. Each circle appears on the screen for 1 second before disappearing. When each circle is displayed, a mouse click is simulated. Since WebGazer is trained on cursor interactions, this simulated mouse click calibrates WebGazer’s gaze-estimation algorithm (Papoutsaki et al., 2016). Once the participant has been presented with all eight calibration circles, a new circle is presented in the middle of the screen for three seconds. During this time, WebGazer calculates the proportion of estimated looks that fall on the center of the screen. In our experiments, calibration was successful if more than 1% of estimated looks fell on the center of the screen. If calibration failed, the calibration procedure would be repeated (no participant failed the WebGazer calibration in either experiment). Note that we purposely selected a low calibration threshold, as we were interested in the variety of calibration scores participants would obtain.^3^

After completing the WebGazer calibration, participants began the task battery. This task battery was split in two parts (created in separate experiment projects on PCIbex). The PCIbex projects preloaded the stimulus resources prior to starting the experiment in order to avoid presentation delays during the experiment. By splitting the experiment into two parts, the loading of these resources is split up, reducing the wait time before the participant can begin the experiment. The first part of the experiment contained the fixation task, smooth pursuit task, and lexical fixation task. Once this part was finished, the participant could take a brief break while the experimenter set up the second part of the experiment. Participants completed the WebGazer calibration sequence again at the beginning of this second part before continuing with the experimental tasks. The second part of the experiment contained the phonemic cohort task and the anticipatory looking task. The details of the tasks are described below, along with the experiment results (Tasks and Results section).

Each task started with a brief WebGazer calibration check: the participants looked at a circle in the center of the screen for three seconds. If more than 1% of estimated looks fell on this circle, the task started automatically. This was the case for all calibration checks (across participants and experiments). If the calibration check had failed (i.e., less than 1% of estimated looks fell on the center circle), the full WebGazer calibration procedure (described above) would have repeated again.

The lab experiment sessions took approximately 30–45 minutes to complete.

#### Web Experiment.

Participants completed the web experiment while in a Zoom teleconferencing meeting with the experimenter(s). After the participant gave their informed consent, the experimenter started a recording of the Zoom meeting. As in the lab experiment, we made separate recordings of the participant’s face and screen. The experimenter sent the participant a link to the PCIbex experiment using Zoom’s chat function. The participant then opened the link in their web browser and used the Zoom screen sharing function to share the experiment display. The participant received instructions from the experimenter over Zoom on how to hide any Zoom controls and windows from the screen before starting the experiment.

The experiment was completed in fullscreen. After participants completed the first part of the experiment, the experimenter sent them the link to the second part using Zoom’s chat function. The PCIbex procedure followed the same format as in the lab experiment. In the case the participant encountered technical difficulties during the experiment, the experimenter could assist them over the Zoom teleconferencing.

The web experiment sessions, like the lab experiment sessions, took approximately 30–45 minutes to complete.

## DATA PROCESSING AND ANALYSES

### WebGazer Calibration Scores

PCIbex computed seven WebGazer calibration scores for each participant throughout the course of the experiment. Participants completed the full WebGazer calibration procedure at the beginning of each part of the task battery (once before starting the fixation task, and once before starting the phonemic cohort task; see Procedure section). The experiment additionally computed a calibration check at the start of each of the five tasks. For each participant, we calculated the average of their scores from the two calibration procedures as well as the average of their scores from the calibration checks.^4^

The grand mean scores from the WebGazer calibration procedures were 57% in the lab experiment (*SD*: 4%, range: 44–63%) and 68% in the web experiment (*SD*: 11%, range: 40–87%). The grand mean scores from the calibration checks were 49% in the lab experiment (*SD*: 8%, range: 25–62%) and 57% in the web experiment (*SD*: 10%, range: 38–78%). The participant means were reliably higher in the web experiment (as measured by a Welch two sample *t*-test) for both the scores from the calibration procedures (*t*(55) = −6.39, *p* < 0.001) and those from the checks (*t*(71) = −3.94, *p* < 0.001). These differences could result from differences in WebGazer sample rates across the two experiments (see Method Sample Rates section), as fps correlates with WebGazer calibration scores (Kandel & Snedeker, 2024; Slim & Hartsuiker, 2023).

### Gaze Recording Processing

WebGazer gaze estimation started and stopped automatically at the beginning and end of each trial in the experiment. Tobii Pro Studio and Zoom both recorded continuous gaze data throughout the duration of the experiment. Therefore, we needed to chop these continuous recordings into individual trial recordings. We did this by using custom Python scripts created by author Anthony Yacovone. In all five tasks of the experiment, a blue fixation circle was presented in the middle of the screen at the start of each trial. This fixation circle functioned as a prompt for the participant to look at the center of the screen, and it also marked the trial onset in the Tobii Pro Studio and Zoom screen recordings. We used these fixation circles to identify the trial onset and offset times in the screen recordings, which were then aligned with the corresponding eye-tracking data. To obtain the trial onset times, the Python script stepped through each frame of the screen recording and identified when the blue fixation circle appeared on the screen. The offset of each trial was identified by the presentation of the blue circle at the start of the subsequent trial.

We then used these timings to identify the trial timings within the corresponding eye-tracking data. The infrared eye-tracking data for each participant consists of a large dataframe with all recorded gaze locations throughout the duration of the experiment. We split this dataframe into individual trials using the trial timings identified from the Tobii Pro Studio screen recording (R script available on https://osf.io/sk8wu/). The manual eye-tracking data, on the other hand, is a video file of the participant’s face. We used a custom Python script to chop the continuous video recording into short videos of each trial, using the trial timings identified from the Zoom screen recording. These trial videos were then annotated for gaze direction (see Preparing the Data for Analysis section).

### Preparing the Data for Analysis

The WebGazer and infrared-eye-tracking data are recorded as screen coordinates. We categorized each recorded gaze as falling on the center (defined as a square with a width/height of 15% of the screen height in the center of the screen) or on one of the four quadrants of the screen (defined as 50% of the display height and width minus any overlap with the center region).

The manual eye-tracking data were generated by coders who manually annotated the participants’ gaze direction by stepping through each frame of the chopped Zoom trial videos. Coders were naive to trial conditions and target stimulus locations. For each frame, the coder determined whether participants’ gaze was on the center of the screen, the top-left quadrant, the top-right quadrant, the bottom-right quadrant, the bottom-left quadrant, or could not be defined (if the participant was blinking, looking away from the screen, or when the video still was not clear). Frames were presented to the coders in mirror image to facilitate left–right coding. A subset of the collected Zoom video data was annotated by secondary coders in order to assess coder reliability (see Supplementary Materials, section B. Manual Eye-Tracking Inter-Coder Reliability). The agreement between the primary and secondary annotations in the reliability check for this data subset was 94.3% (see Supplementary Materials for a break-down of agreement by experiment and task).

We analyzed gaze locations in bins of 100 ms. For quadrant-based analyses (used in the fixation, lexical fixation, phonemic cohort, and anticipatory looking tasks), a time bin received a value of 1 for a region of interest (quadrant or center) if at least 30% of recorded looks during that bin fell in that region of interest. Otherwise, it received a 0. This binarized variable reflects the nature of eye movements: you either look at a region of interest or not.

### Data Exclusion

#### Participant Exclusion Criteria.

A subset of participants in the experiments (lab: *n* = 12; web: *n* = 22) were instructed to use Zoom’s *optimize for video* function when sharing the experiment display for the screen recording (Procedure section). Recall that these screen recordings were used to chop the continuous webcam video recording into individual trial recordings for hand annotation (Gaze Recording Processing section). However, in some of these recordings, there were delays and asynchronies in the timing of the audio and visual cues that prevented proper identification of trial onset in the webcam video data. These timing issues led to earlier than expected gaze movement patterns in participants’ data (see Supplementary Materials, section C. Manual Eye-Tracking Optimize for Video Data). Crucially, these delays were present in the Zoom screen recordings, but not in the experiment presentation—they were not observed during testing, in the Tobii Pro Studio screen recordings, and in the stimulus presentation times recorded by PCIbex. Rather, these timing issues appear to have been caused by adjustments to the recording resolution made as part of Zoom’s *optimize for video* function. Once we noticed issues in the resulting recordings, we stopped using this function; we did not encounter similar stimulus timing issues in any of the screen recordings made after this procedural switch.

The delays in the affected videos were variable, both across participants and at different times within a participant’s recording, making it impossible to align the annotated manual eye-tracking data to the trial onsets. By the time we determined that the video of affected participants would not be able to be aligned, we were unable to collect more lab experiment data due to external constraints. While some participants’ data appeared to be unaffected for some or all tasks (see Supplementary Materials, Figure 2), we took a statistically and empirically conservative approach and omitted the manual eye-tracking data for all participants who used the *optimize for video* function. Any additional participant omissions for each task are described in the Tasks and Results section.

#### Trial Exclusion Criteria.

We omitted trials from the analyses if more than 50% of the recorded looks in the hand-annotated video data for that trial were not directed towards the screen (including blinks, looks away from the experiment screen) or were unidentifiable by the video annotator. This exclusion criterion is intended to omit trials in which participants did not sufficiently attend to the experiment.^5^ Omitting trials based on attention rather than track loss alone allows us to see how well each eye-tracking method performs when participants are looking at the screen.^6^ Any additional, task-specific data exclusions are described in the Tasks and Results section.

In addition to trials omitted from analyses for failing to pass our exclusion criteria, several trials were missing from the WebGazer datasets because there was no corresponding data saved on our data collection server. Such trial loss appears to be inherent to the WebGazer method and has been reported in prior experiments (Kandel & Snedeker, 2024). Consequently, we consider this data loss to be part of the method’s performance and therefore do not similarly omit the missing trials from the infrared eye-tracking or manual eye-tracking datasets. Across all tasks and prior to any participant and other trial exclusions, this affected 0.50% of the trials in the lab WebGazer data and 1.2% of the trials in the web WebGazer data.

### Analysis Procedure

Here we describe the general procedure we used in the analyses of the fixation task, the lexical fixation task, the phonemic cohort task, and the anticipatory looking task (the analysis of the smooth pursuit task followed a different format, see description in Smooth Pursuit Task section). Task-specific variables and analyses are described in the results section of each task in the Tasks and Results section.

The analysis of each task consisted of two parts. First, in the *individual dataset analyses*, we separately analyzed the data for each method (infrared eye-tracking, manual eye-tracking, WebGazer) and experiment (lab, web). Second, we performed *comparison analyses* to assess how effects differed across methods within an experiment setting (the *intra-experiment comparisons*) and within the two webcam methods (manual eye-tracking, WebGazer) across experiment settings (*inter-experiment comparisons*). All analyses were conducted in *R* v4.2.3. (R Core Team, 2023). All analysis models were fit using {lme4} v1.1-34 (Bates et al., 2015). Below we describe the general procedure we used for the individual dataset and comparison analyses.

#### Individual Dataset Analyses.

In the lab experiment, the individual dataset analyses included participants who had analyzable data for all three methods—this allows for maximum comparability between the results of each method in the experiment.

##### Cluster Analyses.

We analyzed the data of each individual method/experiment using cluster-based permutation analyses to test the effect of interest in each dataset. This analysis procedure assesses eye movement patterns over the course of experimental trials (e.g., Hahn et al., 2015; Huang & Snedeker, 2020; Maris & Oostenveld, 2007; Yacovone et al., 2021). We first tested each 100 ms time bin within the analysis time window for our effect of interest, using a logit mixed-effects model (see Tasks and Results section for the analysis window and the variables and model structures used in each task). The effect was considered reliable at a time bin if the analysis yielded a *z*-value with an absolute value greater than 2 (Gelman & Hill, 2006). We then identified clusters of adjacent time bins with reliable effects (in the same direction) and summed the absolute value of the *z*-values of these time bins to obtain a *z*-sum statistic for each cluster.

Next, we assessed the likelihood that any observed clusters would occur by chance by randomly shuffling the relevant condition labels for each participant and testing this permuted dataset for clusters (using the same procedure as above). We repeated this step 1000 times and then compared the *z*-sum statistics obtained in these 1000 simulations to those observed in the original data. If the logit mixed-effects model used in the analysis did not converge (excluding singular fit warnings) or could not be computed at a step (e.g., due to a lack of variation in the dependent variable in one or more cells of the analyses), we did not use the model estimates for that step. Instead, we used the model estimates from the previous time bin, unless the model was at the first time bin, in which case we set the estimates to zero (following Yacovone et al., 2021). The *p*-value is defined as the proportion of *z*-sum statistics that is larger or equal to the *z*-sum statistic observed in the original data (e.g., a *p*-value of < 0.05 indicates that the *z*-sum statistic is less than or equal to 95% of the z-sum statistics in the distribution).^7^ We considered clusters with *p* < 0.05 as significant.

We report the timing of the clusters in milliseconds. For example, if a cluster consisted of the adjacent 100, 200, and 300 time bins, we say that there was a cluster between 100–399 ms.

Note that we cannot make claims about the onset and duration of an effect based on the temporal extent of a significant cluster for three main reasons (for comprehensive discussions, see Fields & Kuperberg, 2020; Groppe et al., 2011; Sassenhagen & Draschkow, 2019). First, the initial step of identifying clusters is purely descriptive, relying on a simple thresholding procedure (i.e., running the test at each time point, and then marking whether the test statistic reached the threshold ∣*z*∣ > 2). Since there are no corrections for multiplicity, there are many opportunities for false positives to emerge. Therefore we cannot infer the true significance of any one time point in the cluster (including the earliest time point). Second, these analyses do not directly address whether including or removing certain time points changes the overall significance of the cluster. Rather, the results of the cluster-mass permutation test simply reports whether the size of a cluster (the *z*-sum) is on the extreme end of an empirical null distribution obtained by looking for clusters in the permuted data. Finally, the temporal extent of a cluster is dependent on the overall power of the study. Thus, the onset and offset of any given cluster is going to be sensitive to the quality of data and may result in an under- or overestimation in the extent of the true effect. Of course, the temporal extent of a significant cluster in the data will be correlated with the true extent of an effect; however, we must be careful in how we interpret these clusters and how we reason about the onset of effects.

##### Effect Size Analyses.

In order to assess differences in the magnitude of gaze patterns detected by the eye-tracking methods, we computed effect size measures for each dataset.^8^ Since there is no straightforward way to calculate effect size metrics for cluster-based permutation analyses due to potential differences in the timings of observed clusters, we calculated three different effect size measures that each highlight different aspects of the effect (following Meyer et al., 2021): the *analysis window effect size*, the *cluster window effect size*, and the *maximum effect size*. These three effect sizes together provide a range in which the true effect size likely lies. The analysis window effect size is the estimated average effect across all time bins that comprise the time window we selected for the cluster-based permutation test. This effect size measure provides a straightforward comparison across datasets, since the timing of the analysis window is precisely defined and the same across data types. However, this effect size is likely an underestimation of the true effect size because it involves time bins with no effect. The cluster window effect size is the estimated effect across all time bins that comprise a cluster. This effect size measure represents the size of the observed effect in the detected cluster. However, because *effects*—and not necessarily clusters—are expected to replicate, this effect size may not replicate. The maximum effect size is the estimated effect in the time bin in which the relevant measure (e.g., difference between the experiment conditions) is the largest. This effect size measure allows us to compare the relevant effect at its peak across datasets. However, this peak effect size likely is an overestimation of the true effect size. Since each effect size measure we estimate comes with a caveat, they should be interpreted together to provide a sense of the true effect size.

For the phonemic cohort and anticipatory looking tasks, we used a Cohen’s *d* score as our effect size metric. To calculate the effect size measures, we fit logit mixed-effects models to the relevant data. These models contained the same random-effects structure as the models used in the individual dataset analyses. The analysis window and cluster window models included an additional random intercept for Time Bin. We then extracted Cohen’s *d* effect size estimates for the model fixed effects using the *lme.dscore()* function of the {EMAtools} R package (Kleiman, 2021). For the fixation and lexical fixation tasks, in which there were no fixed effects in the analysis models, we use the proportion of looks to the target quadrant to create an analogous measure (see fixation task Analyses section for details). In the case that no reliable cluster emerged in a dataset, we only calculated the maximum and analysis window effect sizes for that dataset.

#### Comparison Analyses.

We tested for differences in the time course and magnitude of effects between methods/experiments in the comparison analyses. Datasets were compared in pairs (e.g., for the lab experiment, we compared infrared eye-tracking vs. WebGazer, infrared eye-tracking vs. manual eye-tracking, and WebGazer vs. manual eye-tracking).

In the comparison analyses, we used cluster-based permutation analyses to test for differences in the likelihood of relevant looks across eye-tracking methods (in the *intra-experiment comparisons*) and across experiment settings (in the *inter-experiment comparisons*). The intra-experiment comparisons included the data from all participants with analyzable data of the relevant types (restricting the data across methods to those of the same participants allows for maximum comparability). The inter-experiment comparisons included all of the analyzable data for each method.

The cluster-based permutation analyses followed the same format described above (see Individual Dataset Analyses section). The mixed-models used in this analysis contained the relevant difference as a predictor (Method for the intra-experiment comparisons; Experiment for the inter-experiment comparisons). In the permutation tests for the intra-experiment comparisons, Method was shuffled within participants (e.g., when comparing manual eye-tracking and WebGazer, for each participant, we shuffled which dataset was labeled as manual eye-tracking data and which was labeled as WebGazer data). In the permutation tests for the inter-experiment comparisons, Experiment was shuffled between participants (i.e., we shuffled whether participant datasets were labeled as lab data or web data). These analyses reveal clusters in which there is a difference in the relevant gaze pattern between the compared datasets.

## TASKS AND RESULTS

We present the structure and results for our experiments by task. For each task, we report (i) the task description, (ii) sample size and trial exclusion, (iii) the analyses conducted, and (iv) the results from the analyses. For all tasks (except for the smooth pursuit task, which had a different analysis structure), we report results from the individual dataset analyses, the intra-experiment comparison analyses, and the inter-experiment comparison analyses.

### Fixation Task

#### Task Description.

**Figure 1.**
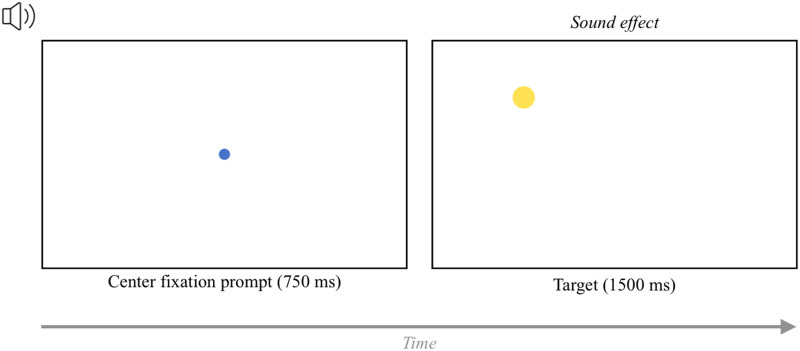
The trial structure of the fixation task. *Note*. This example shows a yellow target stimulus presented in the top-left quadrant. The position of the target stimulus varied across the four quadrants of the screen.

In the fixation task, participants fixated on circles that appear on the screen (e.g., Kandel & Snedeker, 2024; Semmelmann & Weigelt, 2018; Slim & Hartsuiker, 2023). Each trial started with a central fixation, during which a blue fixation circle appeared in the center of the screen for 750 ms. Then, the central circle disappeared and a new circle appeared in the center of one of the four quadrants of the screen (accompanied by a sound effect). This target stimulus remained on screen for 1500 ms (Figure 1). Then, the next trial started automatically.

To break up the repetitive nature of the task, the trials were divided into four blocks. In each block, the target stimulus was a different color (yellow, orange, red, burgundy) and was accompanied by a different sound. Within each block, the target stimulus appeared in each quadrant twice. Therefore, the task had 32 trials total (8 per block). The order of the trials within each block was randomized.

#### Participant and Trial Exclusions.

One participant was omitted from the lab experiment infrared eye-tracking sample because the infrared eye-tracker did not record their data properly (an issue that was resolved later on in the experiment). The participant was omitted from the individual dataset analyses and from the comparison analyses including the infrared eye-tracking data. This participant’s data was not omitted for any other method.

In the comparison analyses, four trials were excluded from the infrared eye-tracking and lab WebGazer data because more than 50% of looks were recorded as missing in the manual eye-tracking data (our proxy of attention; see Data Exclusion section). One trial was excluded from the web WebGazer data for the same reason.

One further lab trial and nine further web trials were missing from the WebGazer data because no data were saved for them on the data collection server.

#### Analyses.

The task analyses collapsed the data across all blocks—the block structure was only implemented to break the repetitive nature of the task and not to trigger meaningful changes in looking behavior.

##### Individual Dataset Analyses.

In the individual dataset analyses, we modeled when looks were more likely to fall on the *side* of the screen containing the target compared to 50% chance.^9^ These analyses were performed within the time window from 0–1499 ms after target stimulus onset. For each dataset, we conducted two such analyses: one analyzing target side looks along the horizontal axis of the screen and one analyzing target side looks along the vertical axis of the screen. These analyses allow us to assess whether the methods differ in their sensitivity to horizontal and vertical look discrimination.

The cluster-based permutation analyses used a logit mixed-effects model with a dependent variable of Target Side Looks (0, 1) and no fixed effects, allowing for comparison of the dependent variable to 50% chance (represented by the intercept). The model contained random intercepts for Subject and Item, with Item defined by target quadrant location (*top-left*, *top-right*, *bottom-left*, *bottom-right*). We did not include random slopes in the model because there were no fixed effects. The Target Side Looks variable was binarized with a 50% threshold such that a time bin received a value of 1 if at least 50% of recorded looks during that bin fell on the target side.^10^ In the permutations, we randomly shuffled the target side (left vs. right in the horizontal analysis; top vs. bottom in the vertical analysis) assigned to each trial for each participant. For each trial, it was then determined whether the observed looks fell on the new target side.

To estimate the size of the target look effect in each dataset, we used the proportion of looks to the target *quadrant* as our effect size metric. We focus on target quadrant looks instead of target side looks for the effect size estimations (and in the comparison analyses; see below), as target quadrant looks is the typical measure used in visual world tasks. For the cluster window effect size and the analysis window effect size measures, we calculated the grand average proportion of target quadrant looks within the relevant window. Since the cluster permutation analyses analyzed whether looks to the side of the screen differed from chance in the horizontal and vertical directions separately, we used the temporal overlap between the clusters identified by these analyses in which target side looks were greater than chance as the cluster window for calculating target quadrant looks. To determine the maximum effect size, we selected the time bin within the cluster window that had the greatest grand average proportion of target looks.

##### Comparison Analyses.

In the comparison analyses, we analyzed differences in target looks across datasets. These analyses investigated the same time window as the individual dataset analyses (0–1499 ms after target onset), though we analyzed looks to the target quadrant instead of target side looks. The cluster-based permutation analyses used a logit mixed-effects model with a dependent variable of Target Quadrant Looks (0, 1). In the intra-experiment comparisons, the predictor variable was Method (e.g., *infrared eye-tracking* vs. *WebGazer*). In the inter-experiment comparisons, the predictor variable was Experiment (*lab*, *web*). The analysis models contained random intercepts for Subject and Item and random slopes of the predictor variable (Method, Experiment) by Subject and Item. The model structure for the intra-experiment comparison additionally contained a random slope for Method by Subject; the inter-experiment comparison model did not contain an analogous slope for Experiment, as Experiment did not vary within-subjects (Barr et al., 2013).

#### Individual Dataset Analysis Results.

**Figure 2.**
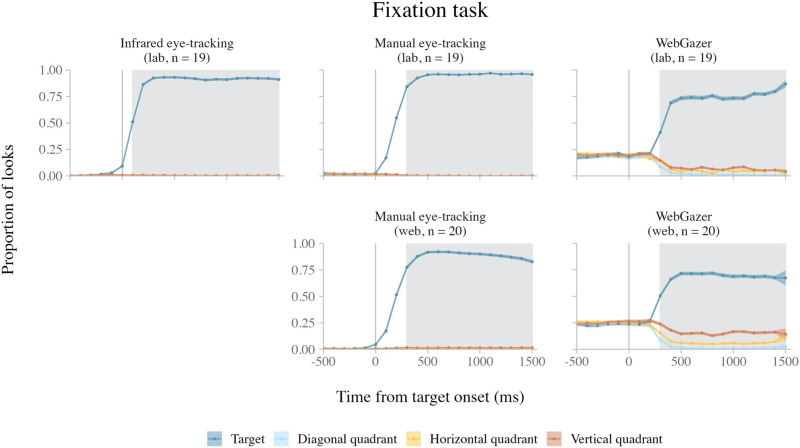
Average looks in the fixation task over time, across methods (columns) and experiments (rows). *Note*. The error ribbons indicate the standard error. The shaded rectangles indicate when target side looks were greater than chance in both the horizontal and vertical direction (see individual dataset analyses).

Figure 2 shows the average looks to the four quadrants of the experimental display over time in each of the five datasets. In all datasets, the proportion of looks to the target quadrant increased after target onset. Table 3 shows the effect size measures for the target quadrant looks in each dataset.

##### Lab Experiment (*n* = 19).

In the infrared eye-tracking data, target side looks were more likely than chance in clusters 100–1499 ms after target onset in both the horizontal (*z*-sum = 77.87, *p* < 0.001) and vertical (*z*-sum = 66.84, *p* < 0.001) directions.

In the lab manual eye-tracking data, target side looks were more likely than chance in clusters 200–1499 ms after target onset in both the horizontal (*z*-sum = 142.03, *p* < 0.001) and vertical (*z*-sum = 144.78, *p* < 0.001) directions. In addition, we observed early clusters showing an effect in the opposite direction: target side looks were *less* likely than chance from 0–199 after target onset in both the horizontal (*z*-sum = 16.27, *p* = 0.015) and vertical (*z*-sum = 18.71, *p* = 0.008) directions.

In the lab WebGazer data, target side looks were more likely than chance in clusters 300–1499 ms after target word onset in both the horizontal (*z*-sum = 54.11, *p* < 0.001) and vertical (*z*-sum = 62.68, *p* < 0.001) directions. In addition, we observed early clusters in which target side looks were less likely than chance from 0–299 ms after target onset in both the horizontal (*z*-sum = 8.79, *p* = 0.014) and vertical (*z*-sum = 9.66, *p* = 0.006) directions.

##### Web Experiment (*n* = 20).

In the web manual eye-tracking data, target side looks were more likely than chance in clusters 300–1499 ms after target onset in both the horizontal (*z*-sum = 79.53, *p* < 0.001) and vertical (*z*-sum = 77.76, *p* < 0.001) directions. In addition, we observed early clusters in which target side looks were less likely than chance from 0–199 ms after target onset in both the horizontal (*z*-sum = 11.94, *p* = 0.017) and vertical (*z*-sum = 12.73, *p* = 0.012) directions.

In the web WebGazer data, target side looks were more likely than chance in clusters 300–1499 ms after target onset in both the horizontal (*z*-sum = 79.88, *p* < 0.001) and vertical (*z*-sum = 56.77, *p* < 0.001) directions.

#### Intra-Experiment Comparison Results.

Focusing first on the intra-experiment comparisons for the lab experiment, target looks were more likely in the infrared eye-tracking data than the lab manual eye-tracking data in a cluster 0–499 ms after target onset (*n* = 19; *z*-sum = 24.81, *p* = 0.001). Target looks were also more likely in the infrared eye-tracking data than in the lab WebGazer data in a cluster 100–1499 ms after onset (*n* = 31; *z*-sum = 76.60, *p* < 0.001). Finally, target looks were more likely in the lab manual eye-tracking data than the lab WebGazer data in a cluster 200–1499 ms after target onset (*n* = 20; *z*-sum = 68.99, *p* = 0.001).

Turning to the web experiment, target looks were more likely in the web manual eye-tracking data than the web WebGazer data in a cluster 200–1499 ms after target onset (*n* = 20; *z*-sum = 39.93, *p* < 0.001).

These results suggest that both infrared eye-tracking and poor man’s eye-tracking detected more target looks than WebGazer throughout the duration of the trial, though infrared eye-tracking was more sensitive to early target looks than manual eye-tracking.

#### Inter-Experiment Comparison Results.

Neither the inter-experiment comparison of the manual eye-tracking data (lab: *n* = 19, web: *n* = 20) nor the inter-experiment comparison of the WebGazer data (lab: *n* = 32, web: *n* = 42) identified any time bin clusters with a significant effect of Experiment, suggesting that the two webcam eye-tracking methods detected target looks with similar resolution in both the lab and web experiments.

#### Summary.

In the fixation task, we tested how well each eye-tracking method discriminated looks to visual cues that appeared on the screen. In both the lab and web experiments, all eye-tracking methods detected an increase in looks to the target quadrant after the target stimulus appeared (Figure 2). In all individual datasets, we observed time windows in which looks to the target were more likely than chance. Nevertheless, there were differences in both the timing and the magnitude of target looks across methods. Infrared eye-tracking detected an earlier increase in target looks than the other two methods, as suggested by the comparison analyses and the cluster timing in the target side look analyses. The lab experiment shows that manual eye-tracking detected a comparable proportion of target looks to infrared eye-tracking (Table 2), although it was less sensitive to early target looks. In both experiments, WebGazer detected the fewest looks of all the methods. During the target fixation windows, both infrared eye-tracking and manual eye-tracking detected ≥89% of looks as falling on the target quadrant, whereas WebGazer only detected 66–69% of looks as falling on the target (Table 2, cluster window effect size). This relatively large spatial error in the WebGazer data also meant that WebGazer was considerably worse than the other methods at detecting looks to the center of the screen; prior to target onset, infrared eye-tracking and manual eye-tracking both detected virtually no looks to any quadrants, while WebGazer detected looks to all quadrants at approximately 25% chance levels (Figure 2) (see Supplementary Materials, section D. Center Looks Identified by Each Method for more detail). This reduction in center looks may have led to relatively earlier discrimination of target side looks in our WebGazer analyses compared to the other methods, as the proportion of target side looks did not start at 0%.

**Table 2.** The Target Look Effect Size Estimates for Each Dataset in the Fixation Task

Experiment	Dataset	Analysis window effect size	Cluster window effect size	Maximum effect size
Lab (*n* = 19)	Infrared eye-tracking	85%	91%	95%
	Manual eye-tracking	81%	92%	97%
	WebGazer	59%	69%	76%
Web (*n* = 20)	Manual eye-tracking	76%	89%	92%
	WebGazer	57%	66%	70%

*Note*. The analysis window effect size reflects the grand average proportion of target quadrant looks for the data within the 0–1499 ms analysis window. The cluster window effect size reflects the grand average proportion of target quadrant looks in the cluster windows (defined as the time window in which the horizontal and the vertical side clusters overlap). The cluster windows used for effect size calculations (measured from target onset) were 100–1499 ms for the infrared eye-tracking data, 200–1499 ms for the lab manual eye-tracking data, 300–1499 ms for the lab WebGazer data, 300–1499 ms for the web manual eye-tracking data, and 300–1499 ms for the web WebGazer data. The maximum effect size reflects the grand average proportion of target quadrant looks within the time bin in the cluster window with the greatest proportion of looks. These three effect sizes together provide a range in which the true effect size likely lies.

Interestingly, web-based testing appeared to add little to no additional noise to the data for the webcam-based eye-tracking methods. The inter-experiment comparisons of both the manual eye-tracking and the WebGazer data detected no differences in target looks across experiments, and the proportions of detected target looks were similar across both experiments for both methods (Table 2).

In sum, all three methods are capable of discriminating looks to regions of interest in quadrant-based experiments in a simple visual fixation task. The main differences are that (i) infrared eye-tracking is the most sensitive of all three methods to early target looks, and (ii) WebGazer is less accurate than the other two methods (independent of setting).

### Smooth Pursuit Task

#### Task Description.

**Figure 3.**
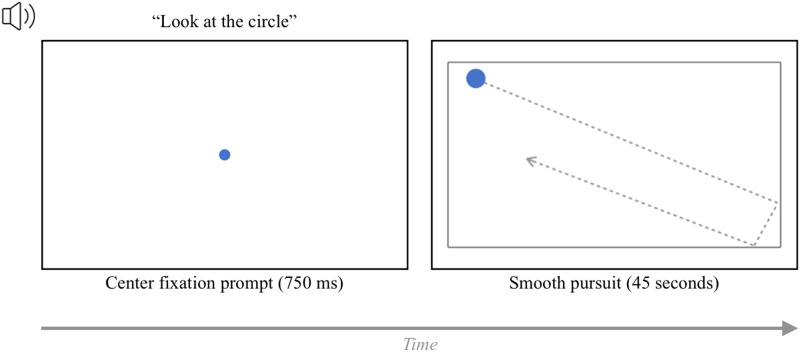
The trial structure of the smooth pursuit task. *Note*. At the start of the task, the participant was prompted to fixate on a circle on the center of the screen. After 750 ms, this central fixation circle disappeared and was replaced by a rectangular box containing a blue circle. The blue circle moved along a random trajectory in this box for 45 seconds. For a video of the full trajectory, please see our OSF repository: https://osf.io/sk8wu/.

In the smooth pursuit task, the participants followed a circle with their gaze as it moved around on the screen. Prior to the task, the participant was prompted to fixate on a circle in the center of the screen. This fixation circle disappeared after 750 ms. Then, the participants followed a blue circle that moved within a black rectangle outline presented in the center of the screen (Figure 3). For all participants, the circle moved in the same trajectory, which was randomly generated using javascript. The task ended after 45 seconds.

#### Participant Exclusion.

One participant was omitted from the infrared eye-tracking sample because the infrared eye-tracker did not record their data properly (an issue that was resolved later in the experiment). One participant was omitted from the lab WebGazer sample because WebGazer only recorded *x*, but not *y*, coordinates of their gaze location. Both participants were omitted from the analyses of the lab experiment data. The participant with the erroneous WebGazer data was further excluded from the inter-experiment comparison analysis.

#### Analyses.

We analyzed the distance offset between the estimated gaze location and the center of the stimulus. For this task, we analyzed infrared eye-tracking and WebGazer data only, because the analysis requires the estimated gaze location to be defined as screen coordinates.

To analyze the data, we first converted the recorded pixel coordinates of the estimated gaze location to a distance metric based on screen size (so the metric was standardized across different screen sizes). The pixel in the center of the screen thus had coordinates (0.5, 0.5), and the pixel in the bottom right corner of the screen had coordinates (1.0, 1.0). Then, we aggregated these data into 100 ms time bins (computing average coordinates for each time bin) and calculated per time bin the Euclidean distance (in screen proportion) between the estimated gaze location and the stimulus location using the formula in (1).

$$\sqrt{{\left(x_{\text{target}}-x_{\text{gaze}\;\text{estimation}}\right)}^{2}+{\left(y_{\text{target}}-y_{\text{gaze}\;\text{estimation}}\right)}^{2}}$$ (1)

We then computed the mean Euclidean distance offset at each time bin for each individual dataset, aggregating over subjects.^11^

We analyzed the data in two ways. First, we conducted an intra-experiment comparison of the infrared eye-tracking data and the WebGazer data for the lab experiment using a paired *t*-test. Second, we performed an inter-experiment comparison of the WebGazer data collected in the lab experiment and the web experiment using a two-sample *t*-test.

#### Results.

**Figure 4.**
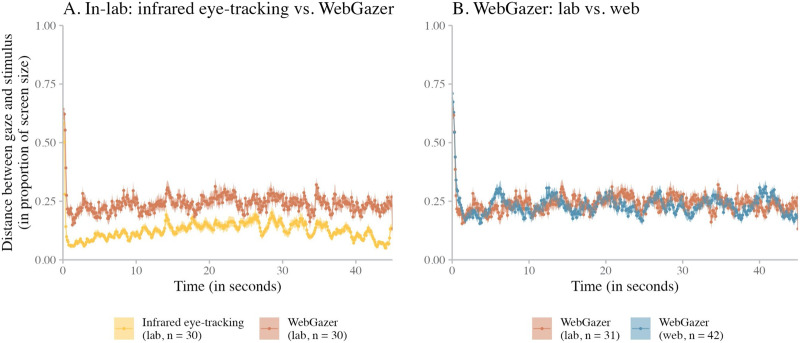
Average Euclidean distance between the estimated gaze location and center of the stimulus over the course of the smooth pursuit task. *Note*. The error ribbons indicate the standard error.

Figure 4 shows the mean Euclidean distance offset at each time bin for each dataset. In all three datasets (infrared eye-tracking, lab WebGazer, and web WebGazer), the data is characterized by an initial, rapid decrease in the Euclidean distance between gaze and the stimulus, which then remains relatively stable over time.

In the lab experiment (*n* = 30), the grand mean Euclidean offset between the estimated gaze position and the stimulus throughout the task was 13% of the screen size (*SD*: 10%) in the infrared eye-tracking data and 24% of the screen size (*SD*: 14%) in the WebGazer data. The intra-experiment comparison analysis (*n* = 30) revealed that the difference between these two datasets was significant (*t*(29) = −9.74, *p* < 0.001).

In the web experiment (*n* = 42), the grand mean Euclidean offset between the estimated gaze position and the stimulus throughout the task was 24% of the screen size (*SD*: 14%) in the WebGazer data. The inter-experiment comparison analysis of the WebGazer data (lab: *n* = 31, web: *n* = 42) revealed that there was no significant difference between the WebGazer data collected in the lab and over the web (*t*(67) = 0.43, *p* = 0.680).

#### Summary.

In the smooth pursuit task, we assessed the offset between the gaze locations estimated by the tested eye-tracking methods (infrared eye-tracking and WebGazer) and the true stimulus location. The gaze locations estimated by the infrared eye-tracker differed from the true stimulus location by approximately 13% of screen size. In contrast, the gaze locations estimated by WebGazer were much larger: approximately 24% of screen size in both the lab and web experiment (there were no reliable differences between the lab and web WebGazer distance offsets in our inter-experiment comparison). In the lab experiment, the same participants’ eye-movements were reliably detected as being closer to the true stimulus location by the infrared eye-tracker than by WebGazer, confirming that infrared eye-tracking provides better spatial resolution when estimating gaze location in a pursuit-style task.

### Lexical Fixation Task

#### Task Description.

**Figure 5.**
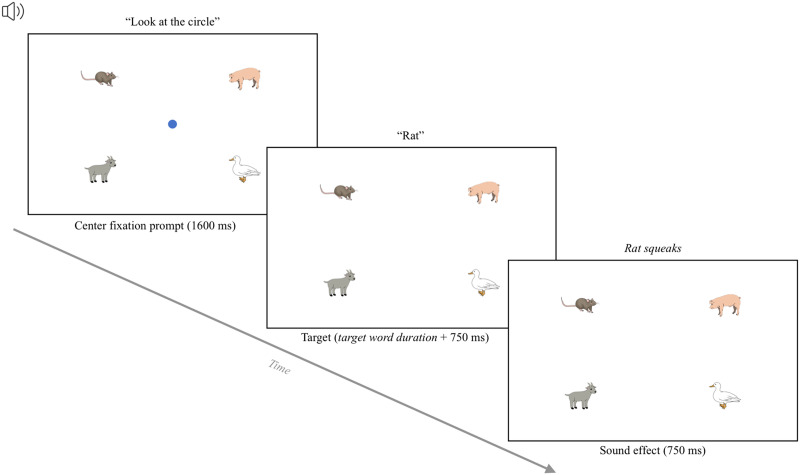
Example lexical fixation trial. *Note*. The same four animals remained in fixed positions throughout the task. In each trial, the participant was first prompted to look at a fixation circle that appeared in the center of the screen. After 1600 ms, this circle disappeared, and the participant was prompted to look at one of the four animals (in this case, the rat). Then, after 750 ms, a sound effect of the target animal was played. The images were taken from www.pixabay.com.

In the lexical fixation task,^12^ participants were prompted by an auditory linguistic cue to fixate on one of four images displayed on the screen. Throughout this task, four animal images (a rat, a pig, a duck, and a goat) were shown in fixed positions on the screen (Figure 5). The images and their locations were the same for each participant and were introduced before the start of the task. In each trial, the participant was prompted to fixate on one of the four animals with a single-word auditory cue (*rat*, *duck*, *goat*, or *pig*). Importantly, all four animals had monosyllabic names that all started with a different consonant, meaning that the disambiguation point within the auditory cue started at cue onset.

Each trial started with 1000 ms of display preview time followed by a central fixation: a blue fixation circle appeared in the center of the screen, together with an auditory prompt to fixate on this circle (*Look at the circle*). Then, 1600 ms after the circle first appeared on the screen, the center fixation circle disappeared, and the participant heard an auditory cue prompting them to look at one of the four animals. At 750 ms after the target word offset, a sound effect of the target animal sound played (Figure 3). The trial then ended 750 ms later.

The task had 16 trials total (4 per animal). The order of the trials was randomized for each participant.

#### Participant and Trial Exclusions.

Two participants were omitted from the web experiment analyses because of issues with the stimulus presentation during the lexical fixation task (possibly due to an unsteady internet connection). Note that one of these participants was already excluded from the manual eye-tracking sample due to data processing issues (see Data Exclusion section).

One trial was excluded from the infrared eye-tracking and lab WebGazer data because more than 50% of looks were recorded as missing in the manual eye-tracking data (our proxy of attention; see Data Exclusion section); the trial was already omitted in the lab manual eye-tracking data.

Two further lab trials and three further web trials were missing from the WebGazer data because no data were saved for them on the data collection server.

#### Analyses.

##### Individual Dataset Analyses.

In the individual dataset analyses, we modeled when looks were more likely to fall on the side of the screen containing the target compared to 50% chance. These analyses followed the same format as the individual dataset analyses for the fixation task (see fixation task Analyses section for details).

As in the fixation task, we used the proportion of looks to the target quadrant to estimate the target look effect size. The effect size calculations followed the same format as in the fixation task.

##### Comparison Analyses.

In the comparison analyses, we analyzed when target looks differed across datasets. These analyses followed the same format as the comparison analyses for the fixation task.

#### Individual Dataset Analysis Results.

**Figure 6.**
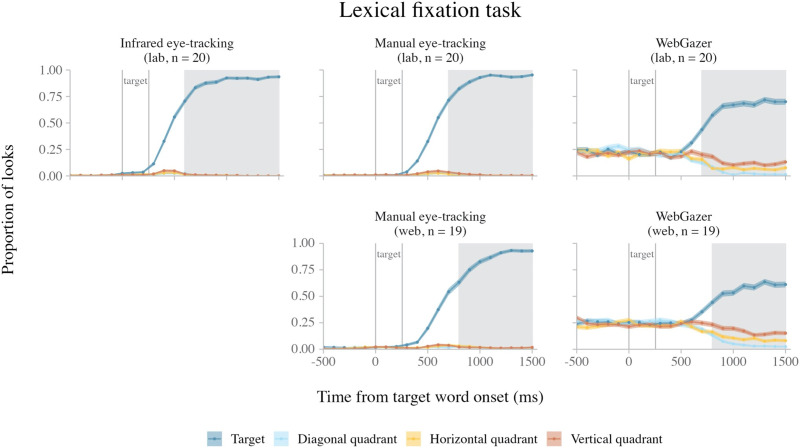
Average looks in the lexical fixation task over time, across methods (columns) and experiments (rows). *Note*. The error ribbons indicate the standard error. Vertical lines indicate average target word duration. The shaded rectangles indicate when target side looks were greater than chance in both the horizontal and vertical direction (see individual dataset analyses).

Figure 6 shows the average looks to the four quadrants of the experimental display over time in each of the five datasets. In all datasets, the proportion of looks to the target quadrant increased after target word onset. Table 3 shows the effect size measures for the target quadrant looks in the individual datasets.

**Table 3.** The Target Look Effect Size Estimates for Each Dataset in the Lexical Fixation Task

Experiment	Dataset	Analysis window effect size	Cluster window effect size	Maximum effect size
Lab (*n* = 20)	Infrared eye-tracking	61%	90%	95%
	Manual eye-tracking	54%	89%	96%
	WebGazer	46%	63%	71%
Web (*n* = 19)	Manual eye-tracking	47%	84%	94%
	WebGazer	40%	56%	64%

*Note*. The analysis window effect size reflects the grand average proportion of target quadrant looks for the data within the 0–1499 ms analysis window. The cluster window effect size reflects the grand average proportion of target quadrant looks in the cluster windows (defined as the time window in which the horizontal and the vertical side clusters overlap). The cluster windows used for effect size calculations (measured from target onset) were 600–1499 ms for the infrared eye-tracking data, 700–1499 ms for the lab manual eye-tracking data, 700–1499 ms for the lab WebGazer data, 800–1499 ms for the web manual eye-tracking data, and 800–1499 ms for the web WebGazer data. The maximum effect size reflects the grand average proportion of target quadrant looks within the time bin in the cluster window with the greatest proportion of looks. These three effect sizes together provide a range in which the true effect size likely lies.

##### Lab Experiment (*n* = 20).

In the infrared eye-tracking data, target side looks were more likely than chance in clusters 500–1499 ms after target word onset in the horizontal direction (*z*-sum = 36.79, *p* < 0.001) and 600–1499 ms after target word onset in the vertical direction (*z*-sum = 30.14, *p* < 0.001). In addition, we observed early clusters along both dimensions showing an effect in the opposite direction: target side looks were less likely than chance from 0–499 ms after target onset in both the horizontal (*z*-sum = 22.85, *p* = 0.004) and vertical (*z*-sum = 26.02, *p* < 0.001) directions.

In the lab manual eye-tracking data, target side looks were more likely than chance in clusters 700–1499 ms after target word onset in both the horizontal (*z*-sum = 54.51, *p* < 0.001) and vertical (*z*-sum = 56.42, *p* < 0.001) directions. In addition, we observed early clusters in which target side looks were less likely than chance from 0–599 ms after target word onset in the horizontal direction (*z*-sum = 41.06, *p* < 0.001) and 100–599 ms after target word onset in the vertical direction (*z*-sum = 30.24, *p* < 0.001).

In the lab WebGazer data, target side looks were more likely than chance in clusters 600–1499 ms after target onset in the horizontal direction (*z*-sum = 50.17, *p* < 0.001) and 700–1499 ms after target onset in the vertical direction (*z*-sum = 39.69, *p* < 0.001).

##### Web Experiment (*n* = 19).

In the web manual eye-tracking data, target side looks were more likely than chance in clusters 800–1499 ms after target word onset in both the horizontal (*z*-sum = 34.37, *p* < 0.001) and vertical (*z*-sum = 34.42, *p* < 0.001) directions. In addition, we observed early clusters in which target side looks were less likely than chance from 0–599 ms after target word onset in both the horizontal (*z*-sum = 38.23, *p* < 0.001) and vertical (*z*-sum = 38.43, *p* < 0.001) directions.

In the web WebGazer data, target side looks were more likely than chance in clusters 600–1499 ms after target onset in the horizontal direction (*z*-sum = 53.43, *p* < 0.001) and 800–1499 ms after target onset in the vertical direction (*z*-sum = 29.79, *p* < 0.001).

#### Intra-Experiment Comparison Results.

Focusing first on the intra-experiment comparisons for the lab experiment, target looks were more likely in the infrared eye-tracking data than the lab manual eye-tracking data in a cluster 300–899 ms after target onset (*n* = 20; *z*-sum = 23.26, *p* < 0.001). Target looks were also more likely in the infrared eye-tracking data than the lab WebGazer data in a cluster 500–1499 ms after target onset (*n* = 32; *z*-sum = 54.19, *p* < 0.001). Target looks were more likely in the lab manual eye-tracking data than the lab WebGazer data in a cluster 600–1499 ms after target onset (*n* = 20; *z*-sum = 39.68, *p* = 0.003). In addition, the comparison analyses identified early clusters from 0–399 ms after target onset in which target looks were more likely in the WebGazer data than in both the infrared eye-tracking data (*z*-sum = 20.18, *p* = 0.015) and the manual eye-tracking data (*z*-sum = 16.62, *p* = 0.015), reflecting the fact that target looks start at 25% chance in the WebGazer data.

Turning to the web experiment, target looks were more likely in the web manual eye-tracking data than the web WebGazer data in a cluster 800–1499 ms after target onset (*n* = 19; *z*-sum = 23.48, *p* = 0.004). In addition, similar to the lab experiment, target looks were more likely in the WebGazer data in an early cluster 0–699 ms after target onset (*z*-sum = 23.15, *p* = 0.004), reflecting that target looks start at 25% chance in the WebGazer data.

These results suggest that both infrared eye-tracking and manual eye-tracking are more sensitive than WebGazer to detect both center looks at the start of the trial as well as target looks during target fixation. As in the fixation task, infrared eye-tracking was more sensitive to early target looks than manual eye-tracking in the lab experiment.

#### Inter-Experiment Comparison Results.

The inter-experiment comparison of the manual eye-tracking data (lab: *n* = 20; web: *n* = 19) identified two time bin clusters with a significant effect of Experiment: One from 400–699 ms after target onset (*z*-sum = 6.43, *p* = 0.045) and one from 800–1199 ms (*z*-sum = 9.67, *p* = 0.020). In these clusters, target looks were more likely in the lab data than in the web data (note: the 700–799 ms time bin separating this cluster nearly reached the significance threshold, with a *z*-score of 1.98). The inter-experiment comparison of the WebGazer data (lab: *n* = 31, web: *n* = 40) did not identify any time bin clusters with a significant effect of Experiment. These results suggest that WebGazer detected target looks with similar resolution in both the lab and web experiments, though target looks were more likely in the lab manual eye-tracking data than the web manual eye-tracking data.

#### Summary.

In the lexical fixation task, we tested how well each eye-tracking method could discriminate target looks guided by linguistic cues. Prior to the onset of the target word, infrared eye-tracking and manual eye-tracking identified virtually no looks to any quadrant, and WebGazer recorded looks to each quadrant at chance (similar to the fixation task; see the fixation task Summary section for discussion). After target onset, all eye-tracking methods tested detected increased looks to the target quadrant (Figure 6), and there were time windows in which looks to the target were more likely than chance.^13^ As in the fixation task, infrared eye-tracking detected more early target looks than the other two methods, as indicated by the cluster onsets in the individual dataset analyses and the intra-experiment comparisons. WebGazer detected the fewest target looks of all the methods, as suggested by the comparison analyses and the effect size estimates: during the target fixation windows, both infrared eye-tracking and manual eye-tracking detected ≥84% of looks as falling on the target quadrant, whereas WebGazer only detected 56–63% of looks as falling on the target (Table 3, cluster window effect size). In contrast, infrared eye-tracking and manual eye-tracking had similar performance, though infrared eye-tracking appears more sensitive to early target looks.

Similar to the fixation task, in the lexical fixation task, setting did not strongly influence the resolution of target looks in the WebGazer data. The inter-experiment analysis found that the likelihood of target looks was higher in the lab manual eye-tracking than in the web manual eye-tracking data, though the proportions of detected target looks were similar in both settings (Table 3).

Thus, all three methods are capable of discriminating looks to quadrant regions of interest in response to linguistic cues. However, infrared eye-tracking appears the most sensitive to early target looks, and WebGazer detects target looks with less accuracy than the other two methods (independent of setting), paralleling the performance of the methods in the fixation task.

### Phonemic Cohort Task

#### Task Description.

The phonemic cohort task was designed to elicit a *phonemic cohort effect* (e.g., Allopenna et al., 1998; Huettig & McQueen, 2007). This effect provides a real-time demonstration of how individuals process the sound components (phonemes) of incoming words and attempt to match them to potential referents. In particular, listeners do not wait until they have heard a full word before they identify it but process the sounds of the word incrementally (Allopenna et al., 1998). Therefore, listeners initially consider all words starting with the same set of sounds (i.e., the spoken word’s *phonemic cohort*) as potential matches to the input and then narrow down to a single word as additional incoming phonemic information disambiguates between the candidate options. In a visual world context, this leads listeners to briefly look at images starting with the same sounds as a spoken word as the word unfolds, only looking away from these images once they’ve received enough sound information to disambiguate its name from that of the target (e.g., looking to a picture of a beetle as they hear the beginning of *beaker*; Allopenna et al., 1998). The phonemic cohort task thus allows us to assess how well the different eye-tracking methods can detect fine-grained real-time processing effects that are short-lived and relatively small (compared to, for example, target fixations).

**Figure 7.**
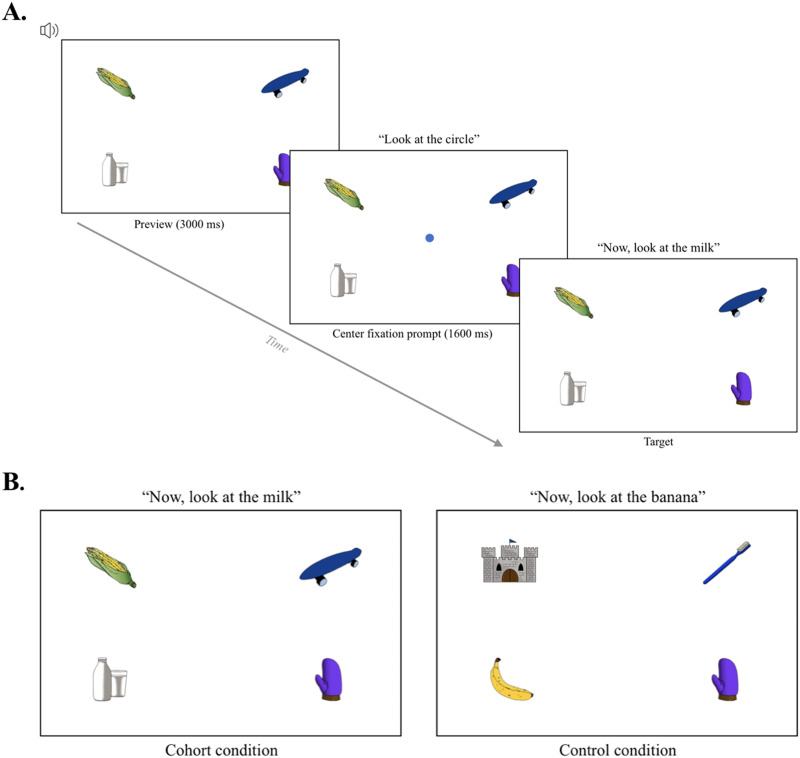
Example phonemic cohort task trial and conditions. *Note*. A: Example phonemic cohort task trial. In each trial, participants saw a different set of images. After 3000 ms of display preview, a central fixation circle was presented on the screen for 1600 ms. Then, the participants were prompted to look at the target image (here, milk). B: Example displays for the competitor image mitten in the cohort condition (left) and the control condition (right). In the cohort condition, the competitor image (mitten) shares the sound onset with the target (*milk*). In the control condition, the same competitor image (mitten) appears in a display with a different target word (*banana*) with which it does not share the sound onset. The figure displays include images from Duñabeitia et al. (2018) and Rossion and Pourtois (2004).

In each trial, the participant was presented with four pictures arranged in quadrants on the screen (Figure 7) and heard a prompt to look at one of the images (the target) (e.g., *Now, look at the milk*). Trials contained a target image (e.g., milk), two distractor images with names unrelated to the target (e.g., skateboard and corn), and a competitor image. The competitor images appeared in two conditions (Figure 7B). In the *cohort* condition, the competitor image shared onset sounds with the target (e.g., *mitten*). In the *control* condition, the competitor image was paired with a target with a different onset (e.g., *banana*). It is predicted that participants look more often at the competitor images in the cohort condition than in the control condition.

Each trial started with a 3000 ms display preview of the four images. Then, a blue fixation circle appeared in the center of the screen accompanied by an auditory prompt to fixate on it (*Look at the circle*). After 750 ms, this central fixation circle disappeared and the participant was prompted to look at the target picture (*Now, look at the [TARGET]*). The trial ended automatically 1500 ms after target word offset.

The phonemic cohort task consisted of 36 trials. In each presentation list, half of these trials were presented in the cohort condition, and the other half in the control condition. Trial order was uniquely randomized per participant.

#### Participant and Trial Exclusions.

There was a programming error in the stimulus presentation for six participants in the lab experiment and eleven participants in the web experiment, which led to the presentation of only cohort (and no control) trials. These participants were omitted from the analysis. Note that these participants were already omitted from the manual eye-tracking samples due to data processing issues (see Data Exclusion section).

One additional participant was omitted from the web manual eye-tracking sample because Zoom did not record their webcam view during the task.

One trial was excluded from the web experiment data because more than 50% of looks were recorded as missing in the manual eye-tracking data (our proxy of attention; see Data Exclusion section).

Four further lab trials and five further web trials were missing from the WebGazer data because no data were saved for them on the data collection server.

#### Analyses.

In the analyses, we analyzed whether participants were more likely to look at the competitor images in the *cohort* condition than the *control* condition, showing evidence of a phonemic cohort effect.

##### Individual Dataset Analyses.

In the phonemic cohort individual dataset analyses, we compared the likelihood of looks to the quadrant containing the competitor image in the *cohort* condition (when the competitor shared onset phonemes with the target) and in the *control* condition (when the competitor and the target had different onsets) within the time window from 0–1499 ms after target word onset (Figure 7B). The cluster-based permutation analyses used a logit mixed effect model with Competitor looks (0, 1) as the dependent variable and Condition (*cohort*, *control*) as the predictor variable. The predictor variable was sum-coded (Hardy, 1993). The models contained random intercepts for Subject and Item, with Item defined by competitor image identity (so, the two displays in Figure 7B are the same item: mitten).^14^

##### Comparison Analyses.

In the phonemic cohort comparison analyses, we analyzed whether the effect of Condition differed across datasets. These analyses investigated the same time window as the individual dataset analyses (0–1499 ms after target onset). The cluster-based permutation analyses used a logit mixed-effects model with a dependent variable of Competitor looks (0, 1). In both the intra- and inter-experiment comparisons, fixed effects were entered into the analysis models using sum-coding (Hardy, 1993). In the intra-experiment comparisons, the predictor variables were Condition (*cohort*, *control*), Method (e.g., *infrared eye-tracking* vs. *WebGazer*), and their interaction; the models contained random intercepts for Subject and Item as well as random slopes for Method by Subject and Item. In the inter-experiment comparisons, the predictor variables were Condition (*cohort*, *control*), Experiment (*lab*, *web*), and their interaction; the models contained random intercepts for Subject and Item and a random slope for Experiment by Item (the model did not have a random slope for Experiment by Subject because Experiment was manipulated between-subjects).

Our analyses focused on the reliability of the interaction term between Method and Condition/Experiment, which indicates whether the detected phonemic cohort effect differs across methods/experiments.

#### Individual Dataset Analysis Results.

**Figure 8.**
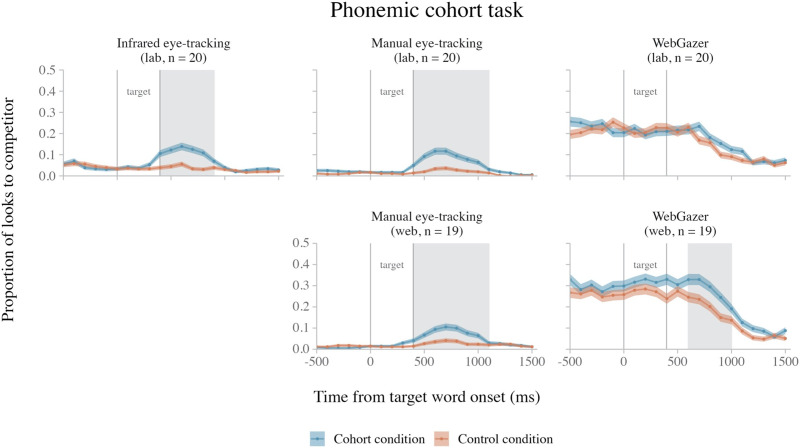
Average looks to the competitor image quadrant in the phonemic cohort task over time (by condition), across methods (columns) and experiments (rows). *Note*. To show the effect more clearly, the y-axis stops at 0.5. Vertical lines indicate average target word duration. The error ribbons represent the standard error. The shaded rectangles mark the cluster windows from the individual dataset analyses when competitor looks were more likely in the cohort condition than the control condition.

Figure 8 shows the average looks to the competitor image quadrant over time in the cohort and control conditions in each of the five datasets. In the individual dataset analyses, we investigated whether the likelihood of looks to the competitor quadrant differed in the cohort and control conditions. Table 4 shows the Cohen’s *d* effect size metrics for the effect of Condition (i.e., the phonemic cohort effect) in each dataset.

**Table 4.** The Phonemic Cohort Effect Size Estimates (Cohen’s *d*) for Each Dataset in the Phonemic Cohort Task

Experiment	Dataset	Analysis window effect size	Cluster window effect size	Maximum effect size
Lab (*n* = 20)	Infrared eye-tracking	0.16	0.31	0.36
	Manual eye-tracking	0.20	0.28	0.34
	WebGazer	0.03	NA	0.17
Web (*n* = 19)	Manual eye-tracking	0.14	0.23	0.26
	WebGazer	0.14	0.21	0.25

*Note*. The analysis window effect size is the estimated effect within the 0–1499 ms analysis window. The cluster window effect size is the estimated effect in the cluster windows. The cluster windows used for effect size calculations (measured from target onset) were 400–899 ms for the infrared eye-tracking data, 400–1099 ms for the lab manual eye-tracking data, 400–1099 ms for the web manual eye-tracking data, and 600–999 ms for the web WebGazer data. We did not calculate the cluster window effect size for the lab WebGazer data since no reliable clusters were identified in the individual dataset analysis. The maximum effect size is the estimated effect within the time bin in the cluster window with the greatest proportion of looks. These three effect sizes together provide a range in which the true effect size likely lies.

##### Lab Experiment (*n* = 20).

In the infrared eye-tracking data, looks to the competitor image were more likely in the cohort condition in a cluster 400–899 ms after target word onset (*z*-sum = 19.10, *p* < 0.001). This cluster indicates a phonemic cohort effect.

Likewise, in the lab manual eye-tracking data, looks to the competitor image were more likely in the cohort condition in a cluster 400–1099 ms after target word onset (*z*-sum = 24.44, *p* < 0.001).

The analysis of the lab WebGazer data, on the other hand, did not identify any time bin clusters with a significant effect of Condition, thus failing to replicate the phonemic cohort effect.

##### Web Experiment (*n* = 19).

In the web manual eye-tracking data, looks to the competitor image were more likely in the cohort condition in a cluster 400–1099 ms after target word onset (*z*-sum = 19.46, *p* = 0.001).

In the web WebGazer data, looks to the competitor image were more likely in the cohort condition in a cluster 600–999 ms after target word onset (*z*-sum = 10.72, *p* = 0.006).

#### Intra-Experiment Comparison Results.

Focusing first on the intra-experiment comparisons for the lab experiment, the comparison of the infrared eye-tracking data and the lab manual eye-tracking data (*n* = 20) did not identify any time bin clusters in which the size of the phonemic cohort effect differed between methods. However, the phonemic cohort effect was larger in the infrared eye-tracking data than in the WebGazer data in a cluster 400–899 ms after target onset (*n* = 26; *z*-sum = 16.87, *p* < 0.001). Likewise, the phonemic cohort effect was larger in the manual eye-tracking data than in the WebGazer data in a cluster 400–899 ms after target onset (*n* = 20; *z*-sum = 14.71, *p* < 0.001).

Turning to the web experiment, the comparison of the web manual eye-tracking data and the web WebGazer data (*n* = 20) did not identify any time bin clusters in which the size of the phonemic cohort effect differed between methods.

These results suggest that in the lab experiment, the size of the phonemic cohort effect was similar in the infrared eye-tracking and in the manual eye-tracking data, and the effects in both these datasets were larger than in the WebGazer data. In the web experiment, on the other hand, the comparison showed no reliable difference between the phonemic cohort effect in the manual eye-tracking and WebGazer data.

#### Inter-Experiment Comparison Results.

Neither the inter-experiment comparison of the manual eye-tracking data (lab: *n* = 20, web: *n* = 20) nor the inter-experiment comparison of the WebGazer data (lab: *n* = 26, web: *n* = 31) identified any time bin clusters with a significant interaction between Condition and Experiment, suggesting that the two webcam eye-tracking methods detected the phonemic cohort effect with similar resolution in both the lab and web experiments.

#### Summary.

In the phonemic cohort task, we assessed whether the three eye-tracking methods under investigation are sensitive enough to detect a spatiotemporally fine-grained real-time language processing effect: the phonemic cohort effect (e.g., Allopenna et al., 1998). In the infrared eye-tracking data and manual eye-tracking data (both lab and web), we observed increases in looks to the competitor images after target word onset in the cohort condition, when the competitor image name began with the same sounds as the target word (Figure 8); no comparable increase was observed for the control condition. Our individual dataset analyses confirmed this phonemic cohort effect, with competitor image looks more likely in the cohort condition than the control condition. The timing of the effects in these datasets is comparable to prior in-lab experiments (e.g., 300–700 ms after target onset; Allopenna et al., 1998). The comparison of the infrared eye-tracking and the lab manual eye-tracking data did not reveal any differences in the size of the phonemic cohort effect between the two methods.

The WebGazer data, however, painted a slightly different picture. In both the lab and web data, looks to the competitor images started at chance and decreased after target word onset (as looks to the target image increased). Visual inspection of the lab and web WebGazer data suggests that this decline is slower in the cohort than in the control condition (indicative of a phonemic cohort effect), though the analyses only detected this difference in the web data. This effect in the web experiment was comparable in size to that detected by manual eye-tracking, as indicated by both the comparison analysis and effect size measurements (Table 4). In contrast, in the lab experiment, infrared eye-tracking and manual eye-tracking detected a larger phonemic cohort effect than WebGazer.

Interestingly, the inter-experiment comparison analyses did not identify any differences in the WebGazer data or in the manual eye-tracking across experiment settings. While this result may be surprising for the WebGazer data (given the differences in the individual dataset analysis), it aligns with the finding observed in previous tasks that setting does not majorly impact the resolution of the data.

Together, these results suggest that infrared eye-tracking and manual eye-tracking consistently pick up the fleeting, spatiotemporally fine-grained phonemic cohort effect, while WebGazer—with its reduced spatiotemporal resolution and increased noise—is less consistent in picking up this small effect.

### Anticipatory Looking Task

#### Task Description.

The anticipatory looking task was inspired by Borovsky et al. (2012) and Altmann and Kamide (1999). The task was designed to elicit *anticipatory looking effects*, which demonstrate that people use sentence context to predict upcoming words as they hear a sentence unfold. In visual world experiments, this leads listeners to fixate on images of words that are likely to appear in a sentence even before they hear the word in the sentence.

**Figure 9.**
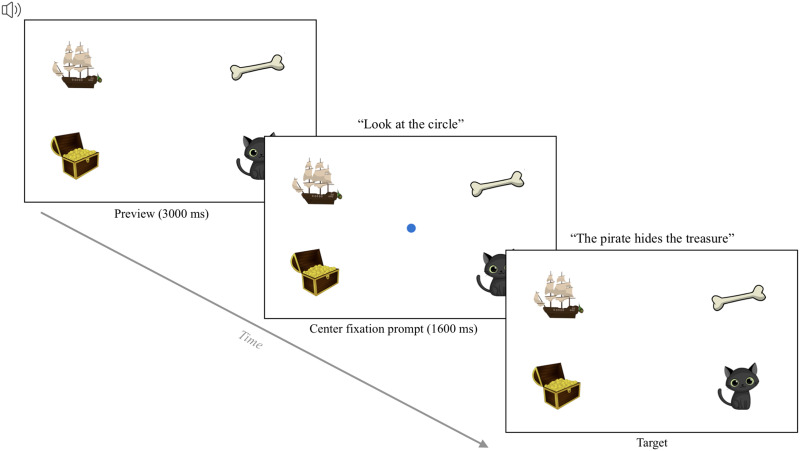
Example anticipatory looking task trial. *Note*. In each trial, participants saw a different set of images. After 3000 ms of display preview, a central fixation circle was presented on the screen for 1600 ms. Then, the fixation circle disappeared and participants heard a test sentence. After the offset of the sentence, participants clicked on the picture that they thought best matched the test sentence. The figure displays include images from pixabay.com.

In each trial, the participants saw a display of four images while they heard a transitive test sentence (in the form *subject* + *verb* + *object*). Participants were asked to click on the picture in the display that was related to the sentence. Each display appeared with an auditory test sentence in one of two conditions (following Altmann & Kamide, 1999). In the *constraining verb* condition, the verb in the sentence was semantically constraining towards a particular post-verbal object (as in *The pirate hides the treasure*). In the *non-constraining verb* condition, this was not the case (as in *The pirate likes the treasure*). Each display contained two images related to the sentence subject (the agent) (e.g., treasure, ship; related to *pirate*) and two images related to an alternative subject (e.g., bone, cat; related to *dog*) (following Borovsky et al., 2012). The two agent-related images were always shown on the same side of the screen (Figure 9). One of the agent-related pictures (the target) was named in the sentence (*treasure*, in this case). This target image was also related to the constraining verb (e.g., *hides*). Test sentences alternated between verb conditions (e.g., *hides* vs. *likes*) and agent/target identity (e.g., *The pirate hides the treasure* vs. *The dog hides the bone*) (balanced across presentation lists).

This design elicits anticipatory looks at two time points in the sentence. First, in both verb conditions, participants are expected to look to the side of the screen containing the agent-related pictures when hearing the sentence subject (the *agent-mediated* anticipatory looking effect; explored descriptively in Borovsky et al., 2012). Second, participants are expected to look at the target image after the verb onset (but prior to target word onset) in the *constraining verb* condition but not in the *non-constraining verb* condition (where target looks are instead predicted to be launched after target onset) (the *verb-mediated* anticipatory looking effect). This is because participants can use verb meaning to pre-emptively identify the target in the constraining verb condition. In our inferential analyses, we focus on the verb-mediated anticipatory looking effect, which is an often-replicated effect in the visual world paradigm (e.g., Altmann & Kamide, 1999; Dijkgraaf et al., 2017; Kamide et al., 2003) that has been previously studied in investigations of webcam-based eye tracking (Ovans, 2022; Slim & Hartsuiker, 2023).

Each trial started with 3000 ms of display preview, after which a blue fixation circle appeared in the center of the screen along with an auditory prompt (*Look at the circle*). After 750 ms, the central fixation circle disappeared and the participant heard the test sentence. After sentence offset, the mouse cursor appeared on the screen, and participants clicked on the image they thought went with the sentence (following Borovsky et al., 2012). After the participants selected an image, they heard a sound effect indicating whether or not their selection was correct, and then the trial ended.

The task consisted of 16 trials total, with half of the trials in the constraining verb condition and the other half in the non-constraining verb condition (counterbalanced across participants). Trial order was fully randomized per participant.

#### Participant and Trial Exclusions.

Two participants were omitted from the web experiment analyses because they did not finish the anticipatory looking task due to internet connectivity issues during testing.

We excluded trials from the analysis in which participants clicked on a picture other than the target: two lab experiment trials and eight web experiment trials were removed for this reason (overall participant accuracy was 99%). An additional five trials were excluded from the lab experiment because more than 50% of looks were recorded as missing in the manual eye-tracking data. Two additional trials were removed from the web experiment (from the same participant) because Zoom did not record the participant’s webcam view during the trial.

One further lab trial and seven further web trials were missing from the WebGazer data because no data were saved for them on the data collection server.

#### Analyses.

In the anticipatory looking task, we analyzed whether participants were more likely to look at the target after hearing the verb (but prior to hearing the target) in the *constraining verb* condition compared to the *non-constraining verb* condition (i.e., the verb-mediated anticipatory looking effect). The agent-mediated looking effect was assessed descriptively through data visualization (following Borovsky et al., 2012).

##### Individual Dataset Analyses.

In the individual dataset analyses, we compared the likelihood of looking at the target image in the *constraining verb* condition and the *non-constraining verb* condition within the time window from 700 ms prior to target word onset to 999 ms after target word onset. The start of this time window is at the mean verb onset relative to the target onset in the test sentences. This time window was chosen because verb-mediated anticipatory looking effects are expected to emerge shortly after participants hear the verb but before they hear the target word. We extended our window after target word onset in order to capture possible delayed evidence of the verb-mediated effects in any of our tested methods (e.g., Slim & Hartsuiker, 2023). The cluster-based permutation analyses used a logit mixed effect model with a dependent variable of Target looks (0, 1) and a fixed effect of Condition (*constraining verb*, *non-constraining verb*). Condition was sum-coded (Hardy, 1993). The models contained random intercepts for Subject and Item, defined here by target identity (e.g., *The pirate hides the treasure* and *The pirate likes the treasure* are the same item).^15^

##### Comparison Analyses.

In the comparison analyses, we analyzed whether the effect of Condition differed across datasets. These analyses investigated the same time window as the individual dataset analyses (−700–999 ms from target word onset). The cluster-based permutation analyses used a logit mixed-effects model with a dependent variable of Target looks (0, 1). In both the intra- and inter-experiment comparisons, fixed effects were entered into the analysis models using sum-coding. In the intra-experiment comparisons, the predictor variables were Condition (*constraining verb*, *non-constraining verb*), Method (e.g., *infrared eye-tracking*, *WebGazer*), and their interaction. The models contained random intercepts for Subject and Item as well as random slopes for Method by Subject and Item.

In the inter-experiment comparisons, the predictor variables were Condition (*constraining verb*, *non-constraining verb*), Experiment (*lab*, *web*), and their interaction. These models contained random intercepts for Subject and Item and a random slope for Experiment by Item (the model did not have a random slope for Experiment by Subject because Experiment was manipulated between-subjects). Analogous to our individual dataset analyses, we did not include slopes for Condition in the random effects of either the intra- or inter-experiment comparison models.

As in the phonemic cohort task, we focused on the reliability of the interaction term, which indicates whether the verb-mediated target looking effect differs across methods/experiments.

#### Individual Dataset Analysis Results.

**Figure 10.**
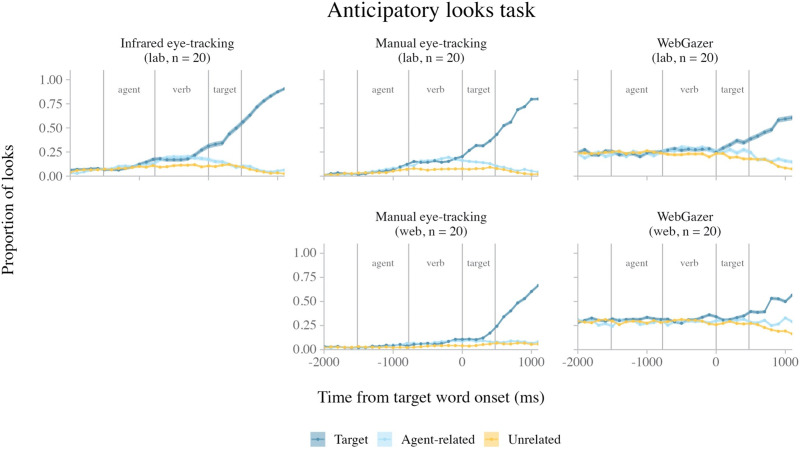
Average looks to the image quadrants in the anticipatory looking task display over time measured from agent word onset (collapsed across verb condition). *Note*. Vertical lines indicate average word durations. The error ribbons indicate the standard error. The images include the target image (depicting the object of the test sentence, related to both the agent and the constraining verb), the agent-related image (the agent-related image that is not the target), and two unrelated distractors (collapsed). Note that we did not conduct inferential analyses of agent-mediated target looks, instead assessing them descriptively (following Borovsky et al., 2012).

Figure 10 shows the average looks to center and the image quadrants in the anticipatory looking task display over time (starting from agent word onset) in each of the five datasets (collapsing across verb condition). The images include the *target* (the agent-related image named in the test sentence), the *agent-related image* (the agent-related image that is not the target), and two *distractors*. For the lab datasets, visual inspection of the plots suggests that looks to the target and agent-related images both increase after agent word onset (although this trend is smaller in the WebGazer data), with looks to the agent-related image then decreasing after verb onset (Figure 10). This pattern is indicative of an agent-mediated anticipatory looking effect. We observe a similar, albeit small, trend in the web manual eye-tracking data, however the agent-mediated anticipatory pattern is not obviously apparent in the web WebGazer data.

**Figure 11.**
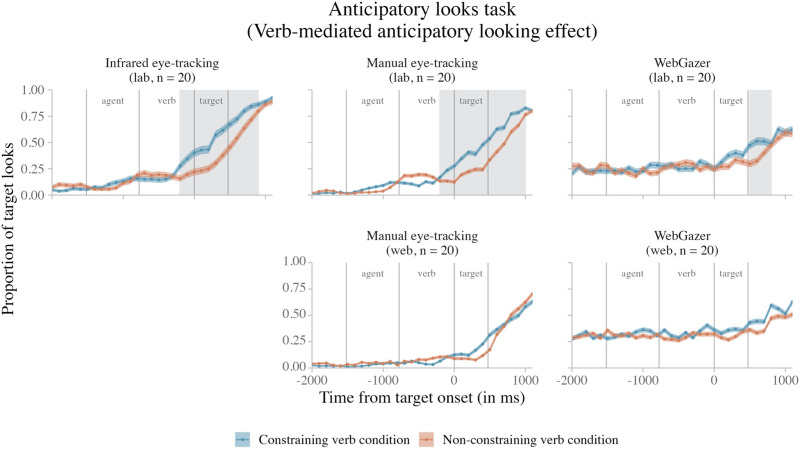
Average looks to the target image quadrant in the anticipatory looking task over time (by verb condition), across methods (columns) and experiments (rows), measured from target word onset. *Note*. The error ribbons indicate the standard error. Vertical lines indicate average word durations. The shaded rectangles mark the cluster windows from the individual dataset analyses when target looks were more likely in the constraining verb condition than the non-constraining verb condition.

Figure 11 shows for each of the five datasets the average looks to the target image quadrant over time in the constraining verb condition and the non-constraining verb condition (the verb-mediated looking effect). In the individual dataset analyses, we investigated when the likelihood of looks to the target image differed in the constraining and non-constraining verb conditions. Table 5 reports the Cohen’s *d* effect size metrics for the effect of Condition (i.e., the verb-mediated looking effect) in each dataset.

**Table 5.** The Verb-Mediated Looking Effect Size Estimates (Cohen’s *d*) for Each Dataset in the Anticipatory Looking Task

Experiment	Dataset	Analysis window effect size	Cluster window effect size	Maximum effect size
Lab (*n* = 20)	Infrared eye-tracking	0.26	0.42	0.64
	Manual eye-tracking	0.20	0.40	0.49
	WebGazer	0.08	0.33	0.40
Web (*n* = 20)	Manual eye-tracking	0.03	NA	0.36
	WebGazer	0.12	NA	0.23

*Note*. The analysis window effect size is the estimated effect within the −700–999 ms analysis window. The cluster window effect size is the estimated effect in the cluster windows. The cluster windows used for effect size calculations (measured from target onset) were −200–899 ms for the infrared eye-tracking data, −200–999 ms for the lab manual eye-tracking data, and 500–799 ms for the lab WebGazer data. We did not calculate the cluster window effect size for the web manual eye-tracking or the web WebGazer data since no reliable clusters were identified in the individual dataset analyses. The maximum effect size is the estimated effect within the time bin in the cluster window with the greatest proportion of looks. These three effect sizes together provide a range in which the true effect size likely lies.

##### Lab Experiment (*n* = 20).

In the infrared eye-tracking data, looks to the target image were more likely in the constraining verb condition in a cluster −200–899 ms from target word onset (*z*-sum = 41.78, *p* = 0.003). In the lab manual eye-tracking data, looks to the target image were more likely in the constraining verb condition in a cluster −100–999 ms from target word onset (*z*-sum = 59.96, *p* < 0.001).^16^ Crucially, these clusters begin prior to target word onset, suggesting that the increase in target looks in the constraining verb condition are predictive looks mediated by the meaning of the verb.

In the lab WebGazer data, looks to the target image were more likely in the constraining verb condition in a cluster 500–799 ms after target word onset (*z*-sum = 9.53, *p* = 0.019). Although this effect is in the expected direction, the cluster emerges after target word onset, making it difficult to identify whether the looks are anticipatory.

##### Web Experiment (*n* = 20).

The analysis of the web manual eye-tracking data did not identify any reliable clusters with a significant effect of verb condition on target image looks.^17^

Likewise, the analysis of the web WebGazer data also did not identify any reliable clusters with a significant effect of verb condition.^18^ Note, however, that in the analysis of the full web WebGazer sample (including participants without analyzable manual eye-tracking data; *n* = 41), looks were more likely in the constraining verb condition in a cluster 200–899 ms after target word onset (*z*-sum = 22, 87, *p* = 0.025).

#### Intra-Experiment Comparison Results.

Focusing first on the intra-experiment comparisons of the lab experiment, the comparison of the infrared eye-tracking data and the lab manual eye-tracking data (*n* = 20) did not identify any time bin clusters in which the size of the verb-mediated looking effect differed between methods. However, the verb-mediated looking effect was larger in the infrared eye-tracking data than in the lab WebGazer data in a cluster 0–499 ms after target onset (*n* = 32; *z*-sum = 13.45, *p* < 0.001), suggesting that the infrared eye-tracking data was more sensitive to early target looks. Conversely, the comparison of the lab manual eye-tracking data and the lab WebGazer data (*n* = 20) did not identify any reliable clusters in which the size of the verb-mediated looking effect differed between methods.^19^

Turning to the web experiment, the comparison of the web manual eye-tracking data and the web WebGazer data (*n* = 20) did not identify any reliable clusters in which the size of the verb-mediated looking effect differed between methods.^20^

These results suggest that in the lab experiment, the size of the verb-mediated looking effect was similar in the infrared eye-tracking and in the lab manual eye-tracking data. Descriptively, the effect seemed more sustained in both these datasets compared to the lab WebGazer data (Figure 11), but the analysis only revealed a reliable difference between the infrared eye-tracking and the lab WebGazer data and not between the manual eye-tracking data and WebGazer data. Likewise, there was no reliable difference between the manual eye-tracking data and WebGazer data in the web experiment.

#### Inter-Experiment Comparison Analysis Results.

Neither the inter-experiment comparison of the manual eye-tracking data (lab: *n* = 20, web: *n* = 20) nor the inter-experiment comparison of the WebGazer data (lab: *n* = 32, web: *n* = 41) identified any time bin clusters with a significant interaction between Condition and Experiment, suggesting that the two webcam eye-tracking methods detected the verb-mediated anticipatory looking effect with similar resolution in both the lab and web experiments.

#### Summary.

In the anticipatory looking task, we examined whether the three eye-tracking methods were able to detect evidence of real-time language processing at the sentence level. In particular, we tested whether participants use contextual cues to predict upcoming words in a sentence, leading them to fixate on images depicting these words before having heard them.

There were two points in our stimulus sentences that could cue participants to launch anticipatory looks. First, the sentence subject (i.e., agent) could cue participants to look at the images on the screen related to this subject in an agent-mediated anticipatory looking effect. We explored this agent-mediated anticipatory looking effect descriptively in Figure 10 (see also Borovsky et al., 2012). In the infrared eye-tracking and manual eye-tracking datasets, looks to both the target image and other agent-related image increased after agent onset, suggesting an agent-mediated anticipatory looking effect (similar to Borovsky et al., 2012). This pattern was reduced or not present in the WebGazer datasets.

The second point in our sentences that could cue anticipatory looks was the verb. We expected participants to look at the target image earlier when the verb was semantically constraining towards the target word as opposed to when the verb was not constraining. We observed differences between the two verb conditions in all datasets of the lab experiment, but the timing of the effect differed between methods. In the infrared and lab manual eye-tracking datasets, the effect emerged in a cluster beginning prior to the target word onset. This is indicative of *anticipatory* processing based on the verb: participants predict upcoming arguments based on the semantics of the verb, which indicates that sentence comprehension is incremental (e.g., Altmann & Kamide, 1999). Comparisons of the infrared eye-tracking and the lab manual eye-tracking furthermore revealed no differences between the observed effects in both datasets, and they showed similar effect sizes (Table 5). In contrast, in the lab WebGazer dataset, the effect of verb condition emerged in clusters beginning well after the target onset—based on this dataset alone, we cannot identify these effects as *predictive* processing (see General Discussion section).

These results suggest that WebGazer is less sensitive than the other methods to the verb-mediated looking effect. This conclusion is supported by the comparison analysis demonstrating that the verb-mediated looking effect was larger in the infrared eye-tracking than the lab WebGazer data in a cluster close to verb onset. Curiously, there were no reliable differences in the comparison between the lab manual eye-tracking and lab WebGazer data, although there was a trending cluster close to verb onset (0–299 ms) suggesting that manual eye-tracking detected a greater effect (*p* = 0.064; see footnote 19).

In contrast to the lab experiment, the web experiment analyses did not reveal reliable verb-mediated looking effects in either the manual eye-tracking or WebGazer datasets. Nevertheless, there is tentative evidence for an influence of verb condition in the web experiment as well. Firstly, there was an effect in the full web WebGazer sample (including participants without manual eye-tracking data). Secondly, visual inspection of the data (Figure 11) suggests a descriptive trend towards verb-mediated looking. Finally, for both the manual eye-tracking and WebGazer datasets, there were unreliable clusters that showed the expected pattern for a verb-mediated looking effect (see footnotes 17–18).

While the method comparisons across the lab and web settings did not detect any reliable differences, the data suggest that there might have been differences between contexts, specific to this task, which resulted in an effect in the lab sample but not the web sample in the individual dataset analyses. In particular, the contrast appears at least in part to derive from differences in gaze patterns across samples. After completion of the experiment, some participants reported that they fixated on the center of the screen until after they had heard the full stimulus sentence. More web than lab participants appeared to exhibit this behavior: *n* = 13 out of *n* = 20 web participants directed ≥75% of looks to the center of the screen during the stimulus sentence in the manual eye-tracking data, compared to *n* = 8 out of *n* = 20 lab participants. We can see the effect of this behavior in Figures 10 and 11, which show delayed onsets of target looks in the web experiment relative to the lab experiment. This behavior may reflect reduced attention in the task or may have been a looking strategy primed by the structure of the preceding tasks, in which participants were explicitly instructed to look at the center until they were told to fixate on a target stimulus. An increase in center looks during the stimulus sentence would correspond to a reduction of anticipatory looks, thereby leading to the absence of a reliable effect in the web sample.

In sum, we find evidence of verb-mediated anticipatory looks in the infrared eye-tracking and lab manual eye-tracking data. In the lab WebGazer data, evidence of a verb-mediated target looking effect was reduced and delayed until *after* target word onset, demonstrating that the method is not well-suited to detecting time-sensitive processing effects. While the verb-mediated looking effects appeared differently in the web experiment, this contrast appears to result from behavioral differences rather than increased noise from the variability of the web-based setting (though we cannot rule out that the setting contributed to these behavioral differences).

## GENERAL DISCUSSION

In this study, we directly compared three eye-tracking methods: (i) infrared eye-tracking, (ii) webcam-based eye-tracking using a manual eye-tracking procedure, and (iii) webcam-based eye-tracking with the automated WebGazer algorithm. In addition, we collected data from the two webcam-based eye-tracking methods in both a lab-based setting and in a (supervised) web-based setting. Across five tasks, we observed that infrared eye-tracking provided data with the highest spatiotemporal accuracy, followed closely by manual eye-tracking. WebGazer was slower to detect eye-movement patterns and did so with a lower spatial accuracy. Finally, web-based testing did not introduce substantial noise to the data.

The present research builds on a growing body of work that investigates the efficacy of webcam-based eye-tracking (e.g., Degen et al., 2021; Kandel & Snedeker, 2024; Ovans, 2022; Semmelmann & Weigelt, 2018; Slim & Hartsuiker, 2023). We expand this literature in several ways. First, we directly compared infrared eye-tracking data, WebGazer data, and manual eye-tracking data recorded simultaneously in the same session, allowing us to isolate the influence of *method* on data quality. In contrast, most previous method comparisons compared data collected across different participant groups and experimental settings (e.g., Semmelmann & Weigelt, 2018; Slim & Hartsuiker, 2023; Vos et al., 2022), and to our knowledge, no prior study includes all three methods. Second, we collected data both in the lab and over the web, allowing us to isolate the influence of experimental *setting* on data quality (see also Semmelmann & Weigelt, 2018). Finally, we tested different effects with a range of effect sizes, which allows us to draw relatively broad conclusions about the use of webcam-based eye-tracking (see also Bogdan et al., 2024; Prystauka et al., 2024).

In the remainder of the General Discussion, we (i) discuss the spatiotemporal accuracy of the different eye-tracking methods under investigation, (ii) assess the consequences for detecting behavioral effects, (iii) present the advantages and disadvantages of each of the tested methods, and (iv) provide recommendations for conducting webcam eye-tracking experiments.

### Spatiotemporal Accuracy of the Eye-Tracking Methods

All three methods detected increases in target looks following the onset of (visual or auditory) cues that directed gaze to the target stimulus. However, they varied considerably in their sensitivity to detect these target looks (Table 6). Infrared eye-tracking and manual eye-tracking detected the greatest proportion of target looks in the fixation and lexical fixation tasks. In contrast, WebGazer detected far fewer target looks. This reduced spatial accuracy was also visible in the smooth pursuit task: the average offset between the estimated gaze locations and the target stimulus was larger in the WebGazer data than in the infrared eye-tracking data by 11% of the screen size.

**Table 6.** Proportion of Target Quadrant Looks by Method in the Fixation and Lexical Fixation Tasks

Task	Sample size	Method	Cluster time window (ms)	Analysis window proportion	Cluster window proportion	Maximum proportion
Fixation	Lab: 19 Web: 20	Infrared eye-tracking	100–1499	85%	90%	95%
		Manual eye-tracking	Lab: 200–1499	Lab: 81%	Lab: 92%	Lab: 97%
			Web: 300–1499	Web: 76%	Web: 89%	Web: 92%
		WebGazer	Lab: 300–1499	Lab: 59%	Lab: 69%	Lab: 76%
			Web: 300–1499	Web: 57%	Web: 66%	Web: 70%

Lexical fixation	Lab: 20 Web: 19	Infrared eye-tracking	600–1499	61%	90%	95%
		Manual eye-tracking	Lab: 700–1499	Lab: 54%	Lab: 89%	Lab: 96%
			Web: 800–1499	Web: 47%	Web: 84%	Web: 94%
		WebGazer	Lab: 700–1499	Lab: 46%	Lab: 63%	Lab: 71%
			Web: 800–1499	Web: 40%	Web: 56%	Web: 64%

*Note*. Cluster time windows are measured from target onset (see task analysis sections for details). The analysis window proportion reflects the proportion of target quadrant looks within the full analysis window (0–1500 ms after target onset). The cluster window proportion reflects the proportion of target quadrant looks within the cluster time window. The maximum proportion reflects the target look proportion within the time bin with the greatest proportion of target looks.

This pattern replicates earlier studies that have shown reduced spatial accuracy in WebGazer data (e.g., Kandel & Snedeker, 2024; Semmelmann & Weigelt, 2018; Slim & Hartsuiker, 2023; *inter alia*) and has consequences for the method’s ability to detect real-time processing effects (see the next subsection for discussion). Moreover, WebGazer appears to lack the spatial resolution to detect looks to smaller regions of interest; although WebGazer discriminated looks to different quadrants, it appeared unable to consistently identify looks to the smaller center region (covering ∼2% of the screen) during center fixations. The infrared and manual eye-tracking data showed virtually no looks to any quadrant when participants were instructed to look to the center of the screen; in contrast, in the WebGazer data, looks to each quadrant was approximately 25%, or at chance levels, suggesting that center looks were not detected (see Supplementary Materials, section D. Center Looks Identified by Each Method).

In addition to the differences in the spatial accuracy of the tested methods, the comparison of the three eye-tracking methods also revealed differences in the *timing* of detected gaze patterns. Firstly, infrared eye-tracking was more sensitive to early looks than manual eye-tracking. Secondly, both infrared eye-tracking and manual eye-tracking appeared more sensitive to early target looks than WebGazer. This pattern is apparent in the visualizations of the fixation and lexical fixation tasks, which show target looks increasing earlier in both the infrared eye-tracking and the manual eye-tracking data compared to the WebGazer data (Figures 2 and 6). This difference is somewhat obscured when we analyze the target looks in each method individually: these analyses suggest target looks are detected with comparable timing across methods (in particular, manual eye-tracking and WebGazer; see the cluster time windows in Table 6). Note, however, that these analyses compared target side looks relative to 50% chance. Since WebGazer starts out with a greater amount of target looks prior to target onset (due to its reduced sensitivity to center looks), a smaller increase of target looks is required to detect an effect in the WebGazer data compared to the other methods. As a result, target looks may have been detected particularly early in the individual WebGazer analyses as a by-product of the way the analysis was conducted. The time lag in the WebGazer data is confirmed by our comparison analyses of the fixation task, where we compared looks to the target quadrants between methods over time. These analyses consistently show that target looks are more likely in the infrared eye-tracking and manual eye-tracking datasets than in the WebGazer dataset early after the target stimulus onset. Moreover, the phonemic cohort task and (lab) anticipatory looking task additionally showed later effects for WebGazer compared to the other two methods. There thus appears to be a delay for WebGazer (as also observed by Degen et al., 2021; Kandel & Snedeker, 2024; Slim & Hartsuiker, 2023; *inter alia*).

Given our within-subjects design, we can rule out an explanation of the apparent WebGazer delay in terms of differences in participant populations. Moreover, this delay cannot be caused by timing issues in the browser-based stimulus presentation software, because our infrared eye-tracking and manual eye-tracking data were collected simultaneously with the WebGazer data, using the same experiment procedure. Finally, we also cannot attribute this delay to differences between lab- and web-based experimentation, because we observed delays in both the lab and in the web experiment. Thus, the most likely explanation for the delay in the WebGazer data is that it is caused by the execution of the WebGazer algorithm itself (see also Kandel & Snedeker, 2024; Semmelmann & Weigelt, 2018; Slim & Hartsuiker, 2023; Yang & Krajbich, 2021). As discussed in Kandel and Snedeker (2024), an apparent delay could arise from two non-mutually exclusive sources. First, a time lag may be introduced to the data because WebGazer requires processing time to estimate gaze position. Second, a delay in effect onsets may arise as a side-effect of WebGazer’s poorer spatial resolution; since the effect sizes at the onset of eye-movement patterns are typically smaller, they will be more difficult to detect when there is increased noise in the spatial signal. Therefore, the onset of an effect will be delayed relative to methods with higher signal-to-noise ratio because a larger increase of looks to the relevant stimulus is necessary before this increase is picked up by WebGazer.

Interestingly, even though previous studies have shown that web experimentation can result in noisier data than lab experimentation (Bogdan et al., 2024; Semmelmann & Weigelt, 2018), we observed highly comparable results in the lab and web experiments. The comparison analyses did not reveal any reliable differences in the webcam eye-tracking methods (WebGazer and manual eye-tracking) between experiments. The only reliable difference was in the lexical fixation task, where manual eye-tracking detected slightly more target looks in the lab experiment (though note that there were also differences in the patterns of results of the individual dataset analyses for the phonemic cohort and anticipatory looking tasks; more below). One potential reason why we observe comparable data between our two experiments is that participants in the web-based experiment were required to use Mac computers to complete the experiment, likely reducing the amount of variability in participants’ hardware. Second, our web experiment utilized a *supervised* testing procedure. Having an experimenter present to direct the participant through the pre-experiment set-up ensured that participants were in an optimal position for webcam-based eye-tracking, and the (virtual) supervision of an experimenter may have raised participant engagement in the task relative to unsupervised experiments. The present findings suggest that web-acquired data can be comparable to lab-acquired data if the procedure used in a web experiment closely simulates that of a lab experiment. Therefore, researchers may want to consider using a supervised testing procedure when investigating very small or fleeting effects.

We recognize, however, that most researchers looking into web-based experimentation are especially interested in *unsupervised* testing. To our knowledge, no studies have directly compared the difference in data quality between supervised and unsupervised web-based eye-tracking experiments. In the Supplementary Materials (section H. WebGazer Data Comparison: Supervised vs. Unsupervised Fixation Tasks), we provide an initial attempt to quantify the differences between supervised and unsupervised web-based testing by comparing our WebGazer fixation task data to those of a very similar fixation task by Slim and Hartsuiker (2023), who used WebGazer in an unsupervised setting. The results were overall very similar between experiments, but WebGazer detected slightly greater proportions of target looks in the fixation task of present study than in Slim and Hartsuiker’s (2023) unsupervised task (as assessed via our target look proportion effect size metrics). However, the results of this comparison should be interpreted with caution due to possibly confounding differences between the two experiments (e.g., differences in task instructions, participant population, and participant hardware). Future research should address the influence of supervised vs. unsupervised testing on WebGazer data quality in a more controlled manner as well as assess the influence of experiment supervision on manual eye-tracking of webcam videos.

### Consequences for Detecting Effects

Most important to behavioral scientists is how well eye-tracking methods detect effects of interest. The previous section reported differences in the spatiotemporal accuracy between the methods. A method’s spatiotemporal accuracy has consequences for its suitability to detect effects. We examined our three methods by testing two real-time processing effects in language comprehension: the phonemic cohort effect and the verb-mediated anticipatory looking effect.

Focusing on the phonemic cohort effect, we saw evidence of cohort competition in the infrared eye-tracking data and the manual eye-tracking data, with an increase in looks to the cohort competitor shortly after target onset. The WebGazer datasets, on the other hand, only revealed a reliable phonemic cohort effect in the web experiment (see Supplementary Materials, G. Combined WebGazer Analyses for results combining the lab and web WebGazer data). Given our within-subjects design in which we simultaneously collected data with different methods, we can safely assume that the lack of an effect in the lab WebGazer data was not because participants’ looking behavior was inconsistent with the phonemic cohort effect. Rather, our results suggest that WebGazer is less consistent in picking up this subtle effect due to its relatively high spatiotemporal error.

Focusing on the verb-mediated anticipatory looking effect (which is a more sustained effect), we observed a slightly different pattern. In the web experiment, we did not observe a reliable verb-mediated looking effect in any of the data (potentially due to a difference in participant looking strategy compared to the lab experiment; see anticipatory looking task Summary section for discussion). Therefore, we restrict our discussion here to the lab experiment. In the lab experiment, we detected an effect of verb-mediated predictive looking in the infrared eye-tracking and manual eye-tracking data. There was also an effect of verb constraint in the WebGazer data, but this effect was smaller than that detected by the other methods, and the effect appeared delayed, with the effect cluster beginning well after target word onset. Note again that, given our within-subjects design, we can conclude that participants in the lab experiment looked at the target before the onset of this cluster (based on the infrared eye-tracking and manual eye-tracking data).

If we consider the WebGazer data in isolation, we cannot be confident that the observed looking patterns reflect any *anticipatory* looks cued by the verb because the name of the target picture has already started playing in the test sentence (see also Slim & Hartsuiker, 2023). For example, this pattern could instead support a theoretical description where verb information facilitates the processing of a target noun due to faster integration of verb and argument information in a non-predictive manner. WebGazer’s reduced spatiotemporal accuracy thus affects the theoretical interpretation of results in studies of real-time processing, thereby limiting WebGazer’s suitability to assess novel effects. For a robust and well-documented effect like verb-mediated anticipatory looking (e.g., Altmann & Kamide, 1999; Borovsky et al., 2012; Dijkgraaf et al., 2017), we can likely assume that any observed effects are triggered by verb information—even if relevant differences between conditions emerge after target onset. However, if we were to use WebGazer to test novel effects, such assumptions would be less grounded and therefore any theoretical interpretation of the time course of effects must be made with particular care. In contrast, infrared eye-tracking and manual eye-tracking allow for a more certain interpretation of the time course of effects.

These results indicate that the eye-tracking methods are differentially sensitive to the detection of real-time processing effects. The differences in spatiotemporal accuracy between the methods translate to differences in effect sizes and, by consequence, the number of participants needed to obtain sufficient power. We assessed differences in power between the methods in post-hoc power analyses that tested how many participants would be needed to detect the observed phonemic cohort effects in our study with each eye-tracking method (note, however, that the results of post-hoc power analyses should be interpreted with caution; see Althouse, 2021 for discussion). We focus on the phonemic cohort effect for our power analyses, as it represents a temporally-sensitive, real-time processing effect, and we observed evidence of this effect in the individual dataset analyses of both the lab and web experiments.

In the first step of our power analysis, we calculated the observed phonemic cohort effect for each method. We used all usable data for each method, and given the overall similarity in the results of the lab and web experiments, we combined the lab and web data for the webcam methods. In our analyses, we assessed each method’s performance in a time window around the peak of its detected effect. This procedure allows us to assess the performance of each method when the effect of interest was at its strongest as well as to more easily compare across methods with different temporal resolutions by restricting the analysis to time windows of the same size (note that in this power analysis, we are only interested in the *size*, and not in the *timing*, of the relevant effect). To identify the peak effect for each method, we calculated the effect size in each time bin in the analysis window using the same model structure as the phonemic cohort individual dataset analyses. Then, we used that model to calculate a Cohen’s *d* effect size for each time bin and took the time bin with the largest effect size together with the previous and the subsequent time bins. The effect within these three time bins represented the observed effect in the relevant dataset for our power analysis.

To calculate this effect for each dataset, we fit logit mixed-effect models testing the effect of interest in this time window (the *observed effect models*). We collapsed the data across the three time bins within this window, calculating the proportion of competitor image looks (the dependent variable used in the phonemic cohort task analyses) in each trial for each participant; we then binarized this measure with a threshold of 30%, meaning that a datapoint was coded as ‘1’ when at least 30% of recorded looks fell on the competitor image (this binarization procedure is analogous to that described in Preparing the Data for Analysis section and reflects the dominant looking behavior in the window of interest).^21^ The observed effect models predicted the likelihood of competitor looks (0, 1) with Condition (*cohort*, *control*) as a fixed effect and a maximal random-effects structure in the sense of Barr et al. (2013).^22^ Note that the effects in the observed effect models are likely an overestimation of the true effect, since they are based on the peak effect window, and consequently should not be taken as a measure of the true effect size of a phonemic cohort effect. In the power analyses, our interest is the relative changes in the effect size across methods rather than the true size of the effect under consideration.

We then conducted the power analyses using the *mixedpower()* function from the {mixedpower} package v0.1.0 (Kumle et al., 2021). For each method, we simulated 1000 datasets based on the existing dataset. In each of these 1000 datasets, we assessed whether there was an effect of the same size as observed in the original dataset (as indicated by the beta-coefficient of the observed effect model). Power is then defined as the proportion of significant outcomes in all 1000 simulations (significance was tested using a *z*-value threshold of 2). For each dataset, we repeated this procedure for sample sizes of 20, 30, 40, 50, and 60 participants.

The results of the power analyses are presented in Table 7. The power analyses showed that manual eye-tracking and infrared eye-tracking reached more power with fewer participants than WebGazer. Both the infrared eye-tracking and manual eye-tracking analyses reached the conventional 80% power threshold at a sample size of 30 participants. In contrast, WebGazer only reached this threshold at 60 participants, suggesting that the phonemic cohort effect detected by WebGazer was reduced in size such that approximately twice as many participants would be required to detect it with sufficient power. Furthermore, if we assume this same reduced effect size (defined by the estimates from the WebGazer observed effect model) for the infrared and manual eye-tracking data, the 80% threshold is again achieved with half of the sample size required for WebGazer (estimated infrared eye-tracking power at *n* = 30: 90%, estimated manual eye-tracking power: 91%). Consequently, the results of our power analyses suggest that researchers interested in running webcam eye-tracking experiments may need to recruit approximately twice as many participants when using WebGazer compared to manual eye-tracking.

**Table 7.** The Estimated Power to Detect the Phonemic Cohort Effect at Various Sample Sizes for All Three Methods

Method	Observed *n*	Observed *β*-estimate (*SE*)	Observed *p*-value (∣*z*∣-value)	Estimated power at *n* =				
				20	30	40	50	60
Infrared eye-tracking	26	0.38 (0.16)	0.020 (2.32)	69%	98%	99%	99%	100%
Manual eye-tracking	39	0.59 (0.17)	<0.001 (3.55)	75%	90%	99%	100%	100%
WebGazer	57	0.16 (0.06)	0.005 (2.78)	37%	54%	64%	73%	83%

*Note*. The windows used to calculate the observed effects were 600–899 ms after target onset for the infrared eye-tracking data, 500–799 after target onset for the manual eye-tracking data, and 700–999 ms after target onset for WebGazer.

Altogether, our results suggest that when selecting an eye-tracking method to use, researchers should consider both (i) the temporal sensitivity and size of the effect under investigation and (ii) available resources for testing. Our results indicate that infrared eye-tracking and manual eye-tracking have better resolution for detecting both large and small effects and require comparable sample sizes for sufficient power. However, as mentioned in the previous subsection, infrared eye-tracking has a slight advantage to detecting early changes in looks, which could be important for very short-lasting, fleeting effects. In contrast, while WebGazer is able to discriminate larger gaze patterns like target looks, the method may struggle to reliably detect more subtle, temporally-fleeting eye-tracking effects (like the phonemic cohort effect) due to its reduced spatiotemporal resolution. Even at the peak of its detected effects, this phonemic cohort effect is smaller, and therefore less-powered, when using WebGazer than when using infrared or manual eye-tracking, and more participants are required to achieve comparable levels of power to the other two methods. Furthermore, effects that are closely time-locked to a (short) time window in the stimulus presentation may be detected by WebGazer with a delay that has consequences for theoretical interpretations (as in the anticipatory looking task; but cf. Prystauka et al., 2024).

### Advantages and Disadvantages of the Eye-Tracking Methods

The results of the current study suggest that the three eye-tracking methods tested have different advantages and disadvantages, making them suitable for different types of experiments. We provide a detailed summary of the advantages and disadvantages for all three methods in Table 8. Here, we highlight the main differences between the two webcam methods (manual eye-tracking and WebGazer).

**Table 8.** Advantages and Disadvantages of the Three Eye-Tracking Methods

Method	Advantages	Disadvantages
Infrared eye-tracking	Usage: No extensive programming skills are required to operate infrared eye-trackers, as the method has been incorporated into easy-to-use experiment builder software (e.g., Tobii Pro Studio/Lab, SR Research Experiment Builder; Tobii Pro, 2021; SR Research, 2021).	Usage: The method requires specialized equipment and (typically) lab space. Infrared eye-tracking equipment is typically not as easily portable as the other methods. To use infrared eye-trackers, participants must be in the same place as the researcher, which is less convenient for scheduling/running participants and makes it more difficult for researchers to access populations not near their home institutions.
	Gaze estimation: The method has a high spatiotemporal accuracy, making it suitable to detect both large and small effects in real-time. The method can identify looks to regions of interest (e.g., quadrants) as well as produce (very fine-grained) coordinate estimates. Infrared eye-trackers often measure other variables of interest as well, such as microsaccades or pupil diameter (Ehinger et al., 2019). Accuracy can be improved through calibration procedures that are easily implemented using the device or experiment builder software.	
	Data processing: Gaze estimates are produced automatically (with no manual coding time required by the experimenter for data processing). Infrared eye-trackers operate with consistent sampling resolution. The method does not save recordings of the participant’s face (maintains participant privacy, reduces data sensitivity, requires less storage space). Infrared eye-trackers provide information about track loss (when the participant was blinking or looking away from the screen).	
Manual eye-tracking	Usage: The method works with off-the-shelf equipment. The experiment itself can be run in any software. There are many ways to implement manual eye-tracking in web-based settings. These implementations typically do not require much specific software from the participant or extensive knowledge on the part of the researcher. The required equipment is easily portable, allowing researchers to set up mobile labs wherever they can bring a laptop. When manual eye-tracking is implemented using teleconferencing software, it is also possible to record the screen that displays the stimuli. These recordings can be used to confirm whether stimuli presentation went as intended.	Usage: When using teleconferencing software (e.g., Zoom) to record videos, it can be more difficult to run experiments in unsupervised web-based settings (although this may be possible with additional instructions; see Slim et al., 2022).
	Gaze estimation: The method identifies looks to regions of interest with a high spatiotemporal accuracy, making it suitable to detect both large and small effects in real-time. Annotators can detect changes based on very subtle eye movements.	Gaze estimation: The method only provides region of interest information; it cannot be used to obtain gaze coordinate estimates.
	Data processing: Depending on how the webcam videos were collected, these videos typically provide consistent sampling resolution (e.g., Zoom standardizes recordings at 25 fps). It is possible to obtain detailed information about track loss because it is visible in the data whether participants were blinking or looking away from the screen.	Data processing: Poor man’s eye-tracking involves time-intensive hand annotations to process the data for analysis. It can be difficult to time-lock recordings to trials onsets, especially in web-based settings. The method requires recording identifiable participant data (in the form of videos), and these video files can require extensive (and sometimes costly) storage space.
WebGazer	Usage: WebGazer is open-source and free to use. The method works with off-the-shelf equipment (a consumer-grade computer, webcam) and runs in freely available internet browsers. The method has integrations with several popular online experiment platforms (e.g., PCIbex, jsPsych, Gorilla, OpenSesame), making it easy to implement web-based eye-tracking experiments. WebGazer can easily be incorporated into web-based experiments in both supervised and unsupervised settings. The required equipment is easily portable, allowing researchers to set up mobile labs wherever they can bring a laptop.	Usage: Some WebGazer implementations require comfort with coding languages (e.g., PCIbex, jsPsych). Not all online experiment platforms come with ready-to-use data collection servers to store WebGazer data (e.g., PCIbex, jsPsych OpenSesame). In that case, the researcher needs to set up their own server, which requires some technical know-how.
	Gaze estimation: The method can identify looks to regions of interest (e.g., quadrants) as well as produce coordinate estimates. WebGazer has built-in calibration procedures that improve accuracy.	Gaze estimation: The method has reduced spatiotemporal accuracy (especially for coordinate estimates), making it less suitable for detecting small and temporally-sensitive effects (particularly in the case of novel effects).
	Data processing: Gaze estimates are produced automatically (with no manual coding time required by the experimenter for data processing). The method does not save recordings of the participant’s face (maintains participant privacy, reduces data sensitivity, requires less storage space).	Data processing: WebGazer has inconsistent sampling resolution. There can be inconsistencies in saving results to data collection servers. It is not obvious how WebGazer treats track loss. WebGazer itself cannot provide any validation of whether the experiment looked and functioned as intended (e.g., by creating a screen recording), though lack of such validation is often a property of unsupervised web-based testing in general.

Our results suggest that manual eye-tracking detects gaze movement patterns with a higher spatiotemporal accuracy than WebGazer. Manual eye-tracking appears capable of detecting large and small effects similarly to infrared eye-tracking (but see Ovans, 2022). Moreover, manual eye-tracking is easy to implement in web-based settings with little technical knowledge on the part of the researcher or the participant, and the experiment itself can be run in any software (e.g., the researcher can use Zoom to screenshare a video or slide deck presentation and record participant eye gaze). However, an important disadvantage of manual eye-tracking is that it requires time-intensive hand annotations (Kandel & Snedeker, 2024 estimate that annotating seven seconds of video takes approximately one minute), and it can be difficult to time-lock recordings to trial onsets.

WebGazer, on the other hand, eases the data processing burden on the researcher. Gaze estimates are produced automatically by the WebGazer algorithm, and gaze recording can be set to start and stop automatically at the beginning and end of each trial. In addition, the method does not require researchers to save identifiable participant video data, thereby decreasing the sensitivity of the stored data, helping maintain participant privacy, and reducing the data storage burden on the part of the researcher. However, WebGazer has inconsistent sampling resolutions and has a much lower signal-to-noise ratio than manual eye-tracking. WebGazer’s reduced spatiotemporal accuracy makes it less suitable for detecting small and temporally-sensitive effects—especially when those effects are novel. Nevertheless, it is worth noting that modifications and updates to the WebGazer algorithm could improve its spatiotemporal accuracy (Yang & Krajbich, 2021, for example, improved WebGazer’s timing by omitting parts of the WebGazer source code, see also Prystauka et al., 2024; Vos et al., 2022; though it is not obvious that these adjustments also improve spatial accuracy).

The version of WebGazer we used performs best at detecting large and long-lasting eye-movement patterns and is thus better suited to experiments where the primary measure of interest is where participants look as opposed to the time course of those movements. In order to detect more sensitive effects, WebGazer requires larger sample sizes than the manual eye-tracking and infrared eye-tracking. However, WebGazer can easily be implemented in unsupervised web-based tasks, which are well-suited for collecting larger samples, thereby allowing researchers to more easily overcome this limitation.

### Recommendations for Webcam Eye-Tracking

We have several recommendations for researchers interested in running webcam eye-tracking experiments using either WebGazer or manual eye-tracking of webcam videos. We break down our suggestions into recommendations for experiment design, data collection, and data processing/analysis.

#### Recommendations for Using Webcam-Based Manual Eye-Tracking.

For researchers interested in using webcam-based manual eye-tracking in their experiments, we have the following recommendations.

*Experiment design recommendations*:

- Place stimuli as far away from each other as possible to make it easier for hand annotators to distinguish looks to different stimuli. Note that this advice differs from that for other eye-tracking methods, which are generally less accurate at estimating gaze locations to the edges of the screen (e.g., Ehinger et al., 2019; Slim & Hartsuiker, 2023).
- Researchers should not use manual eye-tracking if they need coordinate estimates or to discriminate looks across small areas of the screen (e.g., as is the case in eye-tracking-while-reading experiments or visual search paradigms with a crowded display). As WebGazer does not currently provide a sufficient alternative for such measurements, we recommend that experiments requiring fine-grained spatial data be run with infrared eye-tracking.
- Anecdotally, horizontal distinctions are easier for hand annotators to identify than vertical distinctions, especially when participants complete experiments on rectangular computer monitors (in which the horizontal distance between quadrant-based stimuli is larger than the vertical difference). Thus, researchers should consider placing critical stimuli on different sides of the screen.

*Data collection recommendations*:

- Our results suggest that researchers can recruit similar sample sizes to in-lab infrared eye-tracking experiments, as detected effect sizes are similar across methods, at least for supervised web-based experiments (see Table 7). Nevertheless, we recommend researchers confirm their expected effect size(s) in a pilot study.
- Make sure that the participant (and experimenter, if supervised) has a way to preview the webcam stream so that the participant can position themselves such that their eyes are clearly visible. Showing participants this webcam stream again after break periods can help them to readjust their position if it shifts during the task.
- Position participants centered to the camera with the camera at eye level. When the webcam is positioned at a large angle to the participant’s eyes, it can be more difficult to identify distinctions between looks in the resulting recording.
- Re-center participant gaze before presenting relevant stimuli in each trial, as saccade directions are more easily identified when starting from the center.
- Particularly for unsupervised experiments, consider making a recording of the experiment display in addition to the participant’s webcam feed in order to confirm whether the stimulus presentation looked as intended on each trial. It should be possible to make such recordings using custom javascript code or teleconferencing software; to our knowledge, most experiment building software does not have such functions implemented (at the time of writing).
- Consider including sounds in the experiment that can be used to identify and/or check trial timing in the recorded webcam videos during data processing. If making a continuous video recording of the experiment session, these sounds can be used to identify trial onsets. If using PCIbex webcam video recording or another video recording procedure that starts and stops automatically at the beginning and end of each trial, these sounds can be used to check correct trial onset (and correct onset estimates if necessary). Note that this procedure will not work if participants complete the experiment with the sound off or use headphones.
- In order to increase confidence in aligning data to trial onsets during analysis, we recommend including in each experiment a short, fixation-style task for which there are clear expectations about the timing and location of participant looks (this is particularly relevant when using a continuous video recording throughout the duration of the experiment session). The results of this task can be used to help identify whether there are any issues with trial onset alignments during data processing. It can also help to “calibrate” your data coders (more below).
- If using teleconferencing software to collect webcam video recordings, test the functions and setting available to produce optimal recordings. See Kandel and Snedeker (2024) for specific recommendations about using Zoom for manual eye-tracking.

*Data analysis recommendations*:

- Annotate data by participant. Each participant’s webcam video set-up is slightly different, so acclimating to each participants’ set-up can facilitate identification of gaze direction.
- “Calibrate” your coder for hand annotations by including a trial at the start of the experiment in which participants are instructed to look to each region of interest (ROI). This way, your coder can get a sense of how looks to each ROI appear in the video recording for each participant.

#### Recommendations for Using WebGazer.

We have the following recommendations for using WebGazer (note that recommendations for best practice with WebGazer should be updated as the algorithm continues to evolve).

*Experiment design recommendations*:

- Due to its poorer spatial resolution, WebGazer is not well-suited to tasks with crowded displays and small ROIs. We recommend spacing stimuli and using few, large ROIs (e.g., quadrants or halves of the screen).
- Researchers should consider putting critical stimuli on different halves of the screen (or even simplifying to a two-image display) (see also Kandel & Snedeker, 2024; Slim & Hartsuiker, 2023). Diagonally-placed stimuli might be the easiest for WebGazer to discriminate.

*Data collection recommendations*:

- Researchers should expect effect sizes to be smaller than they would be if measured by an infrared eye-tracker. Sample size should be adjusted accordingly (see, e.g., Table 7).
- WebGazer’s accuracy could be improved by increasing the calibration threshold, though note that higher calibration thresholds also result in higher participant dropout rates (e.g., Slim & Hartsuiker, 2023; Vos et al., 2022). Based on the evidence so far, it is difficult to pinpoint the ideal threshold value. In particular, the size of the effect of interest may influence the level of accuracy required to detect it. We therefore recommend researchers to conduct a pilot study in which they test different calibration thresholds (following Vos et al., 2022).
- WebGazer’s performance is highly dependent on the participant’s positioning in front of the webcam and the lighting in the participant’s environment. Experiments should thus contain clear and elaborate instructions telling the participant to sit centrally in front of the webcam and to make sure that their eyes are visible and well-lit (see Semmelmann & Weigelt, 2018 for example instructions). After break periods, participants should be reminded of these instructions again (potentially in concert with a calibration check; see below).
- To help improve WebGazer accuracy, we recommend having regular calibration checks throughout the experiment. These checks will help to ensure that participants remain in an appropriate position throughout the task. Including multiple full calibration procedures throughout the experiment can help reset WebGazer’s accuracy and prevent it from declining throughout the duration of an experiment (e.g., Vos et al., 2022).

*Data processing/analysis recommendations*:

- Prior to analysis, we recommend binning the data to regularize differences in WebGazer sampling rates. Given the variability in WebGazer sampling, particularly in web-based settings (see Method Sample Rates section), larger time bins (e.g., 100 ms) may be most appropriate.
- Given WebGazer’s tendency to detect effects with delays relative to infrared eye-tracking (see Temporal Accuracy section above), we recommend using analysis methods that do not require specifying an effect time window in advance, such as cluster-based permutation analyses (e.g., Huang & Snedeker, 2020; Maris & Oostenveld, 2007).

## CONCLUSION

We examined the suitability of two webcam-based eye-tracking methods (WebGazer and manual eye-tracking of webcam videos) for use in behavioral research, directly comparing them to each other as well as to infrared eye-tracking. In this direct comparison, we simultaneously collected data with all three methods. The comparison revealed that the two webcam-based methods are differentially suitable for detecting the types of eye-movements relevant for behavioral research. Manual eye-tracking produces results approximate to infrared eye-tracking for both large and small effects. In contrast, WebGazer detects gaze patterns with less accuracy, making it less suitable for detecting spatiotemporally-sensitive effects (see also Kandel & Snedeker, 2024; Slim & Hartsuiker, 2023 for a similar conclusion). Interestingly, the webcam methods perform similarly in both lab-based and (supervised) web-based settings. Nevertheless, the webcam eye-tracking methods have different trade-offs that researchers should consider when selecting an eye-tracking method and whether to run experiments online. We expect that continued improvements to webcam-based eye-tracking techniques will open doors for behavioral researchers to take advantage of the opportunities that web-based research has to offer.

## ACKNOWLEDGMENTS

We would like to thank Madeleine Presgrave for her assistance in this project; Jeremy Zehr and Tobii Pro Support for technical troubleshooting guidance; Antara Bhattacharya, Danielle Novak, Eleanor Muir, Itzel Sanchez, Joanna Lau, Madeleine Presgrave, and Timothy Guest for assistance with data collection and/or manual eye-tracking annotation; Caroline Rowland for sharing her thoughts on the initial draft; our anonymous reviewer for their comments and suggestions; and all of the individuals who participated in this work.

## FUNDING INFORMATION

This work was supported by an internal grant from Harvard University, FAS Psychology. Mieke Sarah Slim gratefully acknowledges financial support from the Fulbright Commission The Netherlands foundation and the Research Foundation – Flanders.

## AUTHOR CONTRIBUTIONS

MSS: Conceptualization, Data curation, Formal analysis, Methodology, Project administration, Software, Visualization, Writing – original draft, Writing – review & editing. MK: Conceptualization, Data curation, Formal analysis, Funding acquisition, Investigation, Methodology, Project administration, Software, Writing – original draft, Writing – review & editing. AY: Conceptualization, Data curation, Formal analysis, Methodology, Resources, Software, Writing – original draft, Writing – review & editing. JS: Conceptualization, Data curation, Methodology, Resources, Supervision, Writing – review & editing.

## DATA AVAILABILITY STATEMENT

Data, analysis code, and Supplementary Materials are available from https://osf.io/sk8wu/.

## Notes

^1^ Since the smooth pursuit task did not assess manual eye-tracking data, the analyses for the lab experiment included all participants with analyzable infrared eye-tracking and WebGazer data.^2^ The analysis of the smooth pursuit task for the web experiment included all participants with analyzable WebGazer data (*n* = 42).^3^ Previous studies have shown that increasing the WebGazer calibration threshold improves data quality, but this also leads to higher drop-out rates (e.g., Slim & Hartsuiker, 2023; Vos et al., 2022). Since we are looking into multiple methods and are interested in the variation across them, we did not set a high WebGazer calibration threshold. Our threshold of 1% ensured that WebGazer was working for each participant.^4^ Two participants in the web experiment were missing calibration check scores for the anticipatory looking task because they were unable to complete the task due to internet connectivity issues while testing.^5^ Note that since the annotations did not distinguish between looks away from the screen and unidentifiable looks, we included both types of looks in our omission criterion. Anecdotally, unidentifiable looks were rare, and thus we believe this measure still serves as a reasonable proxy of attention. Unidentifiable looks also included cases when Zoom did not consistently record the participant’s webcam view (as occurred for one web participant); in these cases, we could not confirm participant attention.^6^ This criteria was applied even to participants with alignment issues in their manual eye-tracking data (see Participant Exclusion Criteria section). In these cases, although the onset of the manual eye-tracking data may not align perfectly with trial onset for each trial (with delays on the millisecond scale), the data still captures the majority of the trial and can thus still serve as a proxy of attention.^7^ Although in this analysis it is possible to produce a *p*-value equal to zero (i.e., when 0% of the permutation *z*-sum statistics were larger than or equal to the observed *z*-sum statistic), we report *p*-values of zero as *p* < 0.001.^8^ We additionally preregistered a comparison analysis testing for differences in effect magnitude between datasets in the clusters identified in the individual dataset analyses. We omit these analyses to avoid redundancy, as they produced largely the same pattern of results but do not easily allow for comparison across effects (unlike standardized effect size measures).^9^ These analyses for the fixation and lexical fixation tasks differ from those described in our preregistration, which investigated target quadrant looks. We changed the structure of the analysis to avoid concerns about dependencies within the data.^10^ This threshold was increased from the 30% threshold described in Preparing the Data for Analysis section because the region of interest is larger than in the other analyses (sides vs. quadrants).^11^ We aggregated the time bins over subjects, as the WebGazer data for some participants contained several empty time bins (because, due to a slow sample rate, no data were recorded in that 100 ms bin). Aggregating the data over subjects results in an equal number of samples per dataset (one per time bin), which was needed to use a paired *t*-test.^12^ Also known as the Response to Auditory Target words (R.A.T.) task. This name is particularly appropriate, as there was a rat on the screen.^13^ Across methods, the onset of target looks was ∼400 ms later than in the fixation task, reflecting the additional time necessary to process the auditory target word cue and locate the appropriate image prior to target fixation.^14^ Note that this model deviates from our preregistration, where we proposed a more complex random effects structure. We were underpowered to conduct the individual dataset analyses using a maximal random-effects structure in the sense of Barr et al. (2013); random effects come at a cost of power (see e.g., Matuschek et al., 2017), and we lost a third of our target sample size due to participant omission (see Participant and Trial Exclusions section). This concern did not apply to the prior tasks, which analyzed target fixations with no experimental manipulation. Our main purpose is not to assess phonemic cohort effects, but rather to see how effects change relatively based on the chosen method. We report the results of the analysis using the maximal random effects structure in the Supplementary Materials (section E. Phonemic Cohort Task Maximal Random Effects Analysis).^15^ As in the phonemic cohort task analysis, this random effect structure was simplified from our preregistered structure given concerns about power (see footnote 16). We report the results of the analysis using the maximal random effects structure in the Supplementary Materials (section F. Anticipatory Looking Task Maximal Random Effects Analysis).^16^ The analysis also identified an *unreliable* cluster between −700 – −201 ms after target onset (*z*-sum = 16.57, *p* = 0.091), with target looks more likely in the *non-constraining* verb condition.^17^ The analysis identified two *unreliable* clusters: one from −400 – −201 ms prior to target onset (*z*-sum = 9.04, *p* = 0.359), and one from 300–599 ms after target onset (*z* = 12.93, *p* = 0.201). In the first cluster, the trend is in the opposite direction of verb-driven anticipatory looks—that is, there is a higher likelihood of looks to the target picture in the non-constraining verb condition than in the constraining verb condition. The second cluster shows a trend in the expected direction of verb-based anticipatory looking, with target looks more likely in the constraining verb condition.^18^ The analysis identified two *unreliable* clusters: one from 200–399 ms after target onset (*z*-sum = 5.92, *p* = 0.434) and one from 500–899 ms after target onset (*z*-sum = 11.05, *p* = 0.166). Both these clusters show a trend of verb-based anticipatory looking, with target looks more likely in the constraining verb condition. Note that these two clusters are separated by a single non-significant time bin (400–499 ms; *z* = 0.71).^19^ The analysis identified two *unreliable* clusters with an interaction between Condition and Method. There was an unreliable cluster from 0–299 ms after target onset (*z*-sum = 9.26, *p* = 0.064) in which the verb-mediated looking effect was greater in the manual eye-tracking data. There was also an unreliable cluster from −700 – −401 ms before target onset (*z*-sum = 7.40, *p* = 0.113), however the trend indicated in this cluster does appear to reflect a meaningful difference in verb-mediated looking, as the cluster emerges too early to reflect verb-driven looks, and the trend it specifies is not consistent with a verb-mediated looking effect (target looks were more likely in the non-constraining verb condition than the constraining verb condition in the WebGazer data but not the manual eye-tracking data).^20^ The analysis identified two unreliable clusters with an interaction between Condition and Method: one from −400 – −201 ms before target onset (*z*-sum = 8.85, *p* = 0.225) and one from 400–599 ms after target onset (*z*-sum = 5.09, *p* = 0.478). In the first cluster, the manual eye-tracking data detected a greater difference between conditions than the WebGazer data, however the trend indicated in this cluster is not consistent with a verb-mediated looking effect (target looks were more likely in the non-constraining verb condition than the constraining verb condition). In the second cluster, the verb-mediated looking effect was greater in the manual eye-tracking data.^21^ This collapsing procedure allowed us to avoid complications of pseudoreplication, sparse levels for random effects (if we were to include a random effect of Time Bin), and the potential autocorrelation of residuals that may distort simulation results and lead to inflated power estimates.^22^ This maximal random-effects structure differs from the individual dataset analyses for the phonemic cohort task. The individual dataset analyses for this task used a simpler random effects structure in the cluster analyses due to power concerns. In the present analyses, we are able to use a more complex random effects structure because the analysis collapses across experiment settings and uses all analyzable data for each method, resulting in larger, better-powered datasets.

## Supplemental Materials

https://osf.io/sk8wu/
